# Retinoblastoma protein activity revealed by CRISPRi study of divergent Rbf1 and Rbf2 paralogs

**DOI:** 10.1093/g3journal/jkae238

**Published:** 2024-10-04

**Authors:** Ana-Maria Raicu, Patricia Castanheira, David N Arnosti

**Affiliations:** Cell and Molecular Biology Program, Michigan State University, East Lansing, MI 48824, USA; Department of Biochemistry and Molecular Biology, Michigan State University, East Lansing, MI 48824, USA; Department of Biochemistry and Molecular Biology, Michigan State University, East Lansing, MI 48824, USA

**Keywords:** Retinoblastoma, CRISPRi, transcription, repression, Drosophila

## Abstract

Retinoblastoma tumor suppressor proteins (Rb) are highly conserved metazoan transcriptional corepressors involved in regulating the expression of thousands of genes. The vertebrate lineage and the Drosophila genus independently experienced an Rb gene duplication event, leading to the expression of several Rb paralogs whose unique and redundant roles in gene regulation remain to be fully explored. Here, we used a novel CRISPRi system in Drosophila to identify the significance of paralogy in the Rb family. We engineered dCas9 fusions to the fly Rbf1 and Rbf2 paralogs and deployed them to gene promoters in vivo, studying them in their native chromatin context. By directly querying the in vivo response of dozens of genes to Rbf1 and Rbf2 targeting, using both transcriptional as well as sensitive developmental readouts, we find that Rb paralogs function as “soft repressors” and have highly context-specific activities. Our comparison of targeting endogenous genes to reporter genes in cell culture identified striking differences in activity, underlining the importance of using CRISPRi effectors in a physiologically relevant context to identify paralog-specific activities. Our study uncovers the complexity of Rb-mediated transcriptional regulation in a living organism, and serves as a stepping stone for future CRISPRi development in Drosophila.

## Introduction

The Retinoblastoma (Rb) tumor suppressor protein is an ancient and highly conserved eukaryotic transcriptional corepressor. While most eukaryotes encode a single Rb protein that mediates all the conserved functions of this protein family, vertebrates encode 3 or more Rb paralogs (Cao *et al*. 2010; Dick and Rubin 2013). The human Rb family of pocket proteins comprises the Rb, p107, and p130 paralogs. Rb proteins share a central pocket domain, through which they bind to E2F transcriptional activators on gene promoters (Zhang and Dean 2001). The Rb interaction with E2Fs leads to repression of genes involved in cell cycle progression. Rb proteins also regulate genes involved in DNA repair, transcription, apoptosis, polarity, signaling, and metabolism (reviewed in Chau and Wang 2003; Dick and Rubin 2013; Nicolay and Dyson 2013; Payankaulam *et al*. 2016; Flores and Goodrich 2022). The broader biological significance of paralogy of the Rb family for regulation of gene expression remains unclear. The Drosophila genus experienced an independent RB gene duplication event that is unique among arthropods (Cao *et al*. 2010; Liban *et al*. 2017; Mouawad *et al*. 2019). The Drosophila-specific gene duplication makes it an excellent system for studies aimed at uncovering the evolutionary significance of Rb paralogy, and determining how gene regulatory tasks are apportioned between Rb family members.

Initial studies of the Drosophila Rb paralogs identified major differences in Rbf1 and Rbf2 function and activity (Stevaux 2002; Dimova *et al*. 2003; Keller *et al*. 2005; Stevaux *et al*. 2005). *rbf1* null flies are larval lethal, but *rbf2* null flies are viable, exhibiting altered fertility and decreased lifespan (Stevaux *et al*. 2005; Mouawad *et al*. 2019). Knockdown (KD) of *rbf1* in Drosophila S2 cells leads to upregulation of hundreds of genes, including cell cycle genes, while *rbf2* KD has minimal effects (Dimova *et al*. 2003). We and others have shown that when assayed on cell cycle promoters, Rbf1 is a stronger repressor than Rbf2 (Stevaux 2002; Wei *et al*. 2015; Mouawad *et al*. 2019). Based on these studies, Rbf1 was deemed the predominant corepressor, and Rbf2 a weaker version of Rbf1, playing supposedly redundant roles in gene regulation. Yet, despite its apparently weaker phenotypes, Rbf2 binds to twice as many genes (∼4,000 total) as Rbf1 in embryos, with an enrichment of mitochondrial and ribosomal protein genes that are specifically bound and regulated by the Rbf2 paralog (Acharya *et al*. 2012; Wei *et al*. 2015; Mouawad *et al*. 2019). Additionally, on certain promoters like *tko* and *cycB*, Rbf2 is a stronger repressor than Rbf1 when tested in reporter assays (Wei *et al*. 2015; Mouawad *et al*. 2020).

Drosophila Rb paralogs have preferential binding to promoter-proximal regions, with a peak at −200 bp from the transcriptional start site (TSS) (Acharya *et al*. 2012; Korenjak *et al*. 2012; Wei *et al*. 2015). The significance of this binding preference remains an outstanding question. Rb proteins may directly target the basal machinery as short-range repressors, or they may be constrained by preferential binding to the E2F transcription factors, which themselves are promoter-associated. The recent mapping of Rb proteins to enhancers and insulators in mammals suggests that additional complexity beyond promoter-proximal activity may exist (Sanidas *et al*. 2022).

To assess Rb function and evaluate differences between family members, pioneering studies relied on mammalian Rb fusions to heterologous DNA-binding proteins (such as GAL4) to recruit them to reporter genes in cell culture. Both overlapping and non-redundant roles for Rb paralogs were identified using these methods (Adnane *et al*. 1995; Bremner *et al*. 1995; Weintraub *et al*. 1995; Luo *et al*. 1998). More recently, the Stark laboratory used GAL4 fusions to Drosophila Rbf1 and Rbf2 paralogs in a high-throughput STARR-seq assay, and found similar but not identical patterns of activity by Rbf1 and Rbf2, suggesting that non-overlapping roles exist, at least on plasmid-borne enhancers (Jacobs *et al*. 2023). Yet, such chimeric proteins have not been tested in a living organism in a native chromatin context to investigate possible differences between repression activities mediated by each Rb paralog.

The emergence of CRISPR interference (CRISPRi) as a method involving fusion of transcriptional regulators to the nuclease dead Cas9 (dCas9) presents a path to study mechanisms of repression by the Retinoblastoma paralogs, as well as for repressors and corepressors in general (Gilbert *et al*. 2013; Qi *et al*. 2013). The CRISPRi method is ideal for recruiting repressors to genomic loci of interest without having to alter the underlying DNA sequence. The ability to design guide RNAs (gRNAs) to any desired locus allows for site-specific targeting, permitting placement of the effector across a locus, which can show effects of distance and differing chromatin environments present at promoters. CRISPRi has been widely used in mammalian cells for selective and reversible repression of gene expression from promoter-proximal regions as well as from distal enhancers, and dCas9-effectors have been successfully employed in diverse cell types as well as in vivo in the mouse (Thakore *et al*. 2015; Liu *et al*. 2017; Kampmann 2018; MacLeod *et al*. 2019; Tian *et al*. 2019; reviewed in Carroll and Giacca 2023). Yet, in model organisms that are ideal for uncovering mechanisms of transcriptional regulation, such as *Drosophila melanogaster*, CRISPRi development has lagged behind studies in the mammalian system. The fly model is amenable to large-scale in vivo screens with visible phenotypes, as has been reported for CRISPRa, or CRISPR activation. Notably, CRISPRa has already been successfully developed and employed in the fly for targeted gene activation not only from promoters, but also from enhancers and in heterochromatic regions, and employed in genome-wide screens, indicating that expression of dCas9 chimeras is inherently possible (Ewen-Campen *et al*. 2017; Jia *et al*. 2018; Viswanatha *et al*. 2018; Sajwan and Mannervik 2019; Ortega-Yáñez *et al*. 2022; Xia *et al*. 2023). Additionally, there exists a genome-wide gRNA library for promoter targeting in Drosophila, which has been developed for CRISPRa, as well as thousands of gRNA-expressing fly lines.

Yet, thus far, CRISPRi in Drosophila has relied mostly on dCas9 as a steric blocker, and has been used to target only a small number of genes, including the *roX* long non-coding RNA loci, the *apterous* enhancer, and several protein-coding genes (Ghosh *et al*. 2016; Huynh *et al*. 2018; Aguilar *et al*. 2023; Raicu *et al*. 2024). The ability of dCas9 to function as an active transcriptional repressor in Drosophila, genome-wide, has not been reported yet. Additionally, the only Drosophila CRISPRi effector that has been reported to date is our concurrent development of dCas9-CtBP effectors to study alternatively spliced isoforms of CtBP on a single locus (Raicu *et al*. 2024). Yet, the existence of the genome-wide Drosophila gRNA library for promoter targeting holds promise for facilitating the development of CRISPRi in this model system to study the context-dependent roles of transcriptional repressors, and more broadly to use for inducible and reversible gene repression, genome-wide.

Given the effectiveness of dCas9 fusions to repressor domains tested in other systems, we created novel dCas9 fusions to the Drosophila Retinoblastoma proteins, Rbf1 and Rbf2, to assess the general effectiveness of these 2 highly conserved eukaryotic repressors on diverse gene targets, and to learn about intrinsic repression properties which allow them to function in a similar or non-overlapping manner. Of key interest was how Rb paralogs function in their native chromatin context, and whether previous reports of overlapping or unique functions were inherent to the repression potential of the proteins themselves, or related to differential recruitment to gene promoters by E2F transcription factors (Acharya *et al*. 2012; Korenjak *et al*. 2012; Wei *et al*. 2015). By using novel dCas9-Rbf1 and dCas9-Rbf2 fusions in vivo in whole flies and in cell culture, we identified some instances where the 2 paralogs have similar effects and others in which one paralog displayed stronger repression. Our study shows that Rb proteins are not promiscuous, dominant corepressors; rather, many times they work as “soft repressors”, modestly dialing down gene expression rather than functioning as an on/off gene expression switch (Mitra *et al*. 2021). Additionally, they are highly sensitive to the promoter on which they act, and appear to function in a short-range manner. We uncovered striking differences in effects between endogenous targeting and targeting reporters in S2 cells. Using our CRISPRi system, we also performed structure-function analysis of Rbf1, revealing that on the *E2F2* and *Mpp6* bidirectional promoter, the pocket domain and instability element (IE) are not necessary for repression activity, as has been reported for many other promoters (Acharya *et al*. 2010; Raj *et al*. 2012a, 2012b). Our identification of context-specific roles of Rb paralogs in vivo points to differences in intrinsic repressive activity shaped by evolutionary changes in each paralog over time. Our analysis of dCas9-Rb effectors in vivo and in cell culture indicates that dCas9-repressor chimeras can be successfully used in the Drosophila model system, and presents an exciting opportunity for researchers to further test these corepressors in additional contexts and in large-scale screens, with the goal of further developing CRISPRi for genome-wide targeting in the fly, and to uncover mechanisms of transcriptional repression by transcriptional corepressors in a living organism.

## Materials and methods

### Cloning of dCas9 constructs

The FLAG-tagged (DYKDDDDK) coding sequence for Rbf1 (referred to as the “Rbf” gene on flybase.org; Jenkins *et al*. 2022) was obtained from the pRbf1 plasmid described previously (Acharya *et al*. 2010). Full length dCas9 (with D10A, H840A substitutions) was obtained from the pcDNA-dCas9 vector (gift from Charles Gersbach; Addgene plasmid #47106; Perez-Pinera *et al*. 2013). The dCas9 coding sequence (with two 5′ FLAG epitope tags) was subcloned into the pRbf1 parent vector, 5′ of the Rbf1 sequence. During cloning, a linker between the 3′ end of dCas9 and 5′ end of Rbf1 was inserted, introducing PacI for simpler downstream cloning of other Rb effectors (linker sequence is 5′-GGCTTAATTAATAGTACC-3′ and the peptide sequence of the linker is GLINST). The dCas9-Rbf1 ORF was removed using 5′ NotI and 3′ XbaI sites and cloned into the pUASTattB vector. The final 5XUAS:dCas9-Rbf1 vector was used to subclone other Rb FLAG-tagged sequences into the Rbf1 site using PacI and XbaI, including Rbf2, Rbf1^ΔIE^, and Rbf1^Δpocket^ (Acharya *et al*. 2010; Mouawad *et al*. 2020). Rbf1^ΔIE^ refers to removal of the IE, made up of residues 728–786 in the C-terminus, and was described previously (Acharya *et al*. 2010). Rbf1^Δpocket^ removes the pocket domain, made up of residues 376–728, and was described previously (Acharya *et al*. 2010). All coding sequences for these effectors were removed from their parent vector using PCR amplification with 5′ PacI and 3′ XbaI sites introduced on either end, and replaced the WT Rbf1 sequence. The dCas9 control was created by removing Rbf1 from UAS:dCas9-Rbf1 using 5′ PacI and 3′ XbaI, and inserting a gBlock containing an identical 3′ FLAG found in Rbf1 into the 3′ end of dCas9. We used FlyBase (release FB2018-01–FB2024-01) to obtain genetic sequence information for cloning purposes.

### Cloning of gRNA constructs

Single gRNAs for the *E2F2/Mpp6* promoter were designed using the Drosophila Research & Screening Center (DRSC) Find CRISPRs tool (www.flyrnai.org/crispr/). Nine gRNAs were designed to span the 1 kb putative promoter (including 5′ UTRs) and selected based on several criteria: off-target score <1, machine learning efficiency score close to 1, and Housden efficiency score >5. The gRNA oligos were obtained from IDT and cloned into the pCFD3.1-w vector (Addgene #123366), cutting with BbsI and inserting the annealed oligos. gRNAs are expressed ubiquitously using the U6:3 promoter.

### Drosophila strains and rearing

Flies were fed on standard lab food (molasses, yeast, corn meal) and kept at room temperature in the lab, under normal dark-light conditions. The *nubbin*-GAL4 fly line was obtained from the Bloomington Drosophila Stock Center (BDSC; #25754), and *traffic jam*-GAL4 was a gift from Sally Horne-Badovinac (can be obtained from DGRC as line #104055) (Supplementary Table 4). GAL4 lines were maintained as a homozygous line with a Chr 3 balancer obtained from BDSC #3704. Homozygous dCas9-effector flies were generated by using the *ϕ*C31 integrase service at Rainbow Transgenic Flies Inc. #24749 embryos were injected with each dCas9-effector construct to integrate into Chr 3 landing site 86Fb. Successful transgenic flies were selected through the mini-white selectable marker expression in-house, and maintained as a homozygous line with Chr 2 balancer (from BDSC #3704). These dCas9-effector flies were submitted to Bloomington Drosophila Stock Center and fly lines are indicated in Supplementary Table 3. *driver*-GAL4 and dCas9-effector flies were crossed to generate double homozygotes (*driver*-GAL4 > UAS:dCas9-Rb). The *nubbin*-GAL4 > UAS:dCas9-effector flies were also submitted to Bloomington Drosophila Stock Center and fly lines are indicated in Supplementary Table 3. *nubbin*-GAL4 > UAS:dCas9-Rbf1 homozygous flies had a notched wing phenotype, consistent with Rbf1 overexpression in the wing (Elenbaas *et al*. 2015). *nubbin*-GAL4 > UAS:dCas9-Rbf2 had a less penetrant notched wing phenotype, and *nubbin*-GAL4 > UAS:dCas9 did not have a wing phenotype. The dCas9-VPR construct was obtained as a fly line from the BDSC (#67055). This dCas9 differs from the dCas9 used in our study, as it has 2 additional point mutations aside from D10A and H840A. gRNA fly lines were created by the DRSC, and obtained from the BDSC (fly line numbers indicated in Table 1). Homozygous *nubbin*-GAL4 > UAS:dCas9-Rb flies were crossed to the DRSC homozygous tandem gRNA flies to generate triple heterozygotes (−/−; *nubbin*-GAL4/gRNA; UAS:dCas9-Rb/+). For single gRNAs designed in this study (Supplementary Table 1), #25709 embryos were injected with each gRNA plasmid through the *ϕ*C31 integrase service at Rainbow Transgenic Flies Inc., to integrate into Chr 2 landing site 25C6, which is the same landing site as the DRSC gRNA lines used here. Successful transgenic flies were selected through the mini-white selectable marker expression in-house, and maintained as a homozygous line. Homozygous *nubbin*-GAL4 > UAS:dCas9-Rb flies were crossed to the homozygous single gRNA flies to generate triple heterozygotes (−/−; *nubbin*-GAL4/gRNA; UAS:dCas9-Rb/+).

**Table 1.** Genes targeted in vivo by dCas9-effectors in this study.

Gene name	Function	gRNA binding site	BDSC #	Phentoype with dCas9-Rbf1	Phenotype with dCas9-Rbf2	Phenotype with dCas9-VPR	Phenotype with dCas9	Rbf1 ChIP peak	Rbf2 ChIP peak
*E2F2*	Cell cycle repressor	−577, −672	78707	x	x			x	x
*Mpp6*	M phase phosphoprotein	−18, + 57	78707	x	x			x	x
*InR*	Insulin receptor	−112, −420	78685	x	x	x	x	x	x
*wg*	Ligand in wnt signaling	−314, −366	67545		x	x	x		
*Acf*	Nucleosome remodeling complex subunit	−42, −228	80174	x	x	x	x	x	x
*Pex2*	Peroxisome protein	−70, −333	78252	x	x	x		x	x
*mcm6*	Helicase subunit involved in DNA replication	−383, −438	79845	x	x	x	x	x	x
*dpp*	Ligand in TGFb signaling	−3, −450	67554	x	x	x	x	x	
*E2F1*	Cell cycle activator	−171, −238	78700					x	x
*DNApol⍺180*	Catalytic subunit of DNAP	−57, −202	78146	x			x	x	x
*Sta*	Ribosomal protein	−72, −302	78627					x	x
*mRpS22*	Mitochondrial ribosomal protein	−364, −476	78159					x	
*Atx2*	Involved in eye development	−213, −476	77320	x		x		x	x
*ct*	Transcription factor		67524		x		x		
*Rbf1*	Retinoblastoma factor 1	−65, −442	80755		x			x	x
*Rbf2*	Retinoblastoma factor 2	−187, −410	79982					x	
*p53*	Transcription factor	−211, −327	80207					x	x
*GstE13*	Glutathione transferase	−244, −471	77283					x	x
*vang*	Establishes planar polarity in epithelia	−69, −332	79671					x	x
*EloB*	Controls wing cell identity	−266, −374	79926						
*Atf3*	Activating transcription factor	−112, −283	80180	x		x			
*Mnn1*	Regulates stress response	−23, −388	80730					x	x
*Wwox*	Oxidoreductase in OXPHOS	−191, −359	77229						x
*chico*	InR substrate	−312, −363	76114						
*spen*	Regulator of wnt signaling	−355, −424	80177						x
*dad*	Inhibitory SMAD in dpp pathway	−294, −398	79923					x	x
*SIN3A*	Coreprepressor	−56, −142	78727						
*L(2)37Cc*	Involved in hypoxia, localizes to mitochondria	−53, −175	78654						x

Twenty-eight genes were selected to be targeted by dCas9-Rb effectors. Gene names are indicated, along with: their function, binding site of gRNAs relative to the flybase.org annotated TSS (gRNAs designed by DRSC), the Bloomington Drosophila Stock Center (BDSC) fly number, whether any phenotype was observed after dCas9-effector targeting (indicated with “x”), and whether Rbf1 and Rbf2 ChIP-seq peaks exist on the gene's promoter based on Drosophila embryo ChIP-seq (indicated with “x”) (Wei *et al*. 2015).

### Genotyping flies

All flies generated in this study were genotyped at the adult stage. Flies of each genotype were homogenized (1 fly/tube) in 50 µl squish buffer (1 M Tris pH 8.0, 0.5 M EDTA, 5 M NaCl with 1 µl of 10 mg/ml Proteinase K for each fly). Tubes were set at 37°C for 30 min, 95°C for 2 min, centrifuged at 14,000 RPM in an Eppendorf Centrifuge 5,430 for 7 min, and stored at 4°C. Following PCR amplification, amplicons were cleaned using Wizard SV-Gel and PCR Clean-Up System, and sent for Sanger sequencing.

### Imaging adult wings and phenotype scoring

Adult wings were collected from ∼50 male and female 1–3 day-old adults that were mated and kept at room temperature. They were stored in 100% ethanol in −20°C until dissection. Wings were removed, mounted onto ASi non-charged microscope slides using Permount, and photographed with a Canon PowerShot A95 camera attached to a Leica DMLB microscope. Images were all taken at the same magnification (10×) and using the same software settings. Phenotypes were assessed and categorized into: supernumerary bristles, ectopic veins, short or missing anterior cross vein (ACV)/posterior cross vein, irregular border, smaller L2–L3 intervein region, and combinations of these phenotypes.

### Wing disc dissections and RT-qPCR

Fifty third instar (L3) wing discs were dissected from L3 larvae and placed in 200 µl TRIzol (Ambion TRIzol Reagent) and stored in −80°C until use. RNA was extracted using chloroform and the QIAGEN maXtract High Density kit, and stored in −80°C. cDNA synthesis was performed using applied biosystems High Capacity cDNA Reverse Transcription Kit. RT-qPCR was performed using SYBR green (PerfeCTa SYBR Green FastMix Low ROX by Quantabio) and measured using the QuantStudio 3 machine by applied biosystems. Three control genes were averaged (*Rp49*, *RpS13*, *CG8636*) for all samples, with control samples obtained from crossing dCas9 or dCas9 effectors to a non-targeting gRNA (QUAS). Primers were designed using the IDT REalTIme qPCR assay tool, and sequences are indicated in Supplementary Table 2. RT-qPCR was performed on 3–4 biological replicates with averages of 2 technical duplicates. Student's *t*-test (2 tailed, *P* < 0.05) was used to measure statistical significance. Error bars indicate SEM.

### Ovary dissections and RT-qPCR

For determining changes in gene expression in the ovary, *traffic jam*-GAL4 > UAS:dCas9-Rb flies were crossed to the DRSC *E2F2* gRNA fly line. Female adult progeny were kept at 27°C with male progeny for 3–4 days, and were fed a normal diet supplemented with yeast. 20–25 ovaries per replicate were dissected in 1X PBS with 0.3% Triton, and were rinsed in cold 1X PBS before transferring to Trizol for dissociation. Samples were frozen at −80°C prior to RNA extraction. RNA was extracted as described above, was immediately treated with DNase (TURBO DNA-free kit) and with RNAse inhibitor during the cDNA synthesis stage. RT-qPCR was performed as described above.

### Cloning of *E2F2*- and *Mpp6*-luciferase reporters

Luciferase reporter constructs were created by amplifying yw^67^ (Mani-Telang and Arnosti 2007) genomic DNA with primers flanking the 1 kb promoter that is shared between *E2F2* and *Mpp6*, including the 5′ UTR of *E2F2* and *Mpp6* but excluding the coding sequences. The promoter was inserted into 5′ NotI and 3′ HindIII sites in the luciferase reporter plasmid described previously (Wei *et al*. 2016) in both orientations, generating *Mpp6*-luciferase (the *Mpp6* ATG is proximal to luciferase TSS) and *E2F2*-luciferase (the *E2F2* ATG is proximal to luciferase TSS) reporters (Wei *et al*. 2015).

### Luciferase reporter assays

*Drosophila* S2 cells were grown in 25°C in Schneider Drosophila medium supplemented with glutamine (Gibco) containing 10% FBS and 1% penicillin-streptomycin. Equal amounts of plasmids were co-transfected into 1.5 million cells with QIAGEN Effectene transfection reagent. For experiments in Fig. 6, 250 ng of each plasmid was used: *E2F2*- or *Mpp6*-luciferase reporter, *actin*-GAL4 (Addgene #24344), 1 UAS:dCas9-Rb plasmid, and 1 sgRNA plasmid targeting the *E2F2/Mpp6* shared promoter. Cells were harvested after 72 h and the luciferase assay was performed using the Biotium Steady-Luc HTS assay kit. Briefly, cell media was removed after centrifugation, and cells were resuspended in 1X PBS. The lysate was used in triplicate (70 µl of lysate added to 70 µl of luciferase reagent) and quantified using the Veritas microplate luminometer (Turner Biosystems). Values were normalized to control wells (expressing an empty pCFD3 plasmid instead of a gRNA plasmid). Three technical replicates were averaged for each biological replicate. Three to 5 biological replicates were compared via Student's *t*-test (2 tailed, *P* < 0.05) to test for significance.

### Western blot

Fifteen L3 wing discs were dissected in 1X PBS and placed in 35 µl cell lysis buffer (50 mM Tris, pH 8.0; 150 mM NaCl; 1% Triton X-100). Tubes were flash frozen in liquid nitrogen and stored in −80°C. Samples were homogenized and protein was extracted by freezing, thawing, and vortexing several times, followed by boiling in 1X Laemmli buffer. Twenty microliters of lysate was separated on a 4–20% resolving gel (Bio-Rad Mini-PROTEAN TGX Precast Gel #456-1094), and transferred to a PVDF membrane for analysis using α-FLAG antibody (Sigma Aldrich #F3165, 1:10,000), and α-CtBP as the loading control (DNA208; Mani-Telang and Arnosti 2007). Blocking with both primary and secondary antibodies was performed in 5% milk-TBST (50 mM Tris-HCl, pH 7.4, 150 mM NaCl, 0.1% Tween 20). Blots were developed using HRP-conjugated GαM and GαR secondary antibody (Pierce), and imaged using SuperSignal West Pico PLUS chemiluminescent substrate.

## Results

### Development of fly lines expressing dCas9-Rb chimeras

**Fig. 1.**
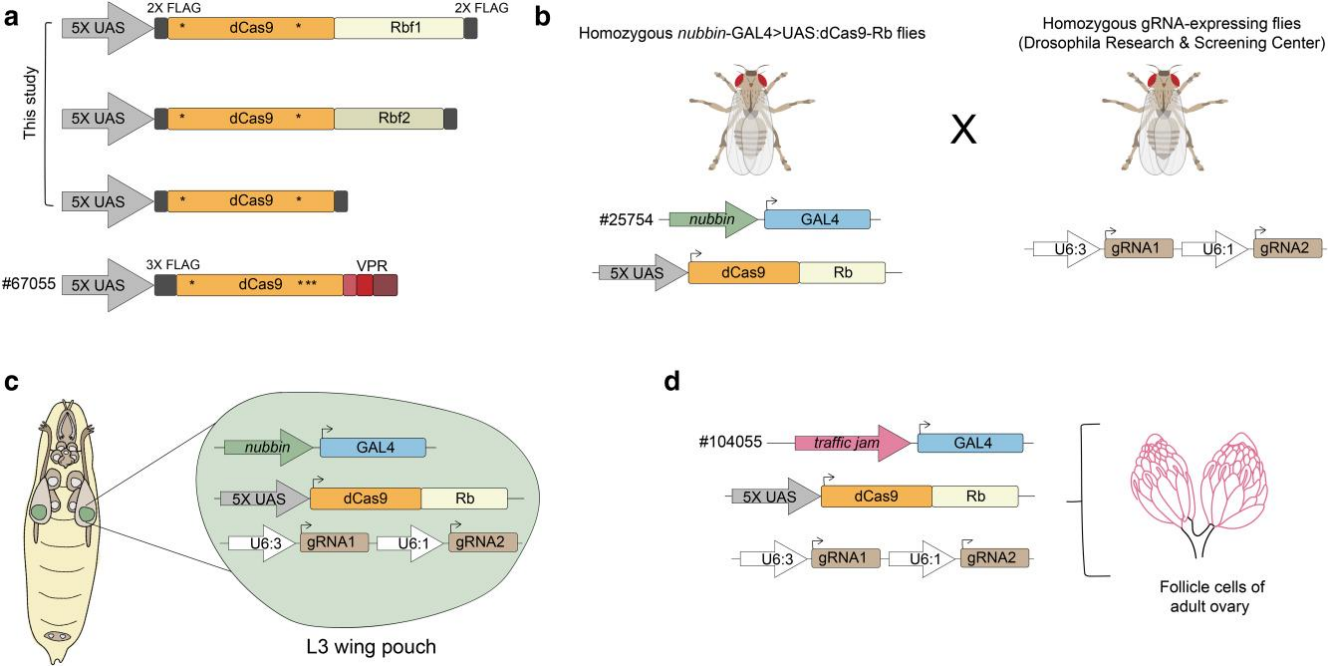
Creation of an in vivo CRISPRi system in Drosophila to target Rb paralogs to endogenous gene promoters. a) The fly Rbf1 and Rbf2 FLAG-tagged coding sequences were fused to the C-terminus of the *S. pyogenes* nuclease dead Cas9 (dCas9; asterisks denote inactivating mutations), and placed under UAS expression. FLAG-tagged dCas9 was created as a negative control. dCas9-VPR was obtained as a fly line (#67055 from BDSC); VPR is a tripartite activator that was previously characterized as a Drosophila transcriptional activator (Lin *et al*. 2015). b) Here, we generated homozygous flies expressing UAS:dCas9-Rb chimeras under the expression of *nubbin*-GAL4 (expression predominantly in the wing pouch of L3 wing discs; Azpiazu and Morata 2000; Wu and Cohen 2002). We crossed these fly lines to homozygous flies expressing 2 gRNAs for a single gene's promoter (from the DRSC). These tandem gRNAs bind within 400 bp of a gene's TSS, and are ubiquitously expressed. c) Progeny from the cross indicated in b) express 3 transgenes in order to target one of the dCas9 chimeras to a single gene's promoter in the L3 wing disc; these flies were used for most of the in vivo experiments described in this study. d) Generation of flies expressing UAS:dCas9-Rb effectors in the follicle cells of the adult female ovary using a *traffic jam*-GAL4 driver (#104055 from the DGRC). Fly crossing scheme is as depicted in b).

**Fig. 2.**
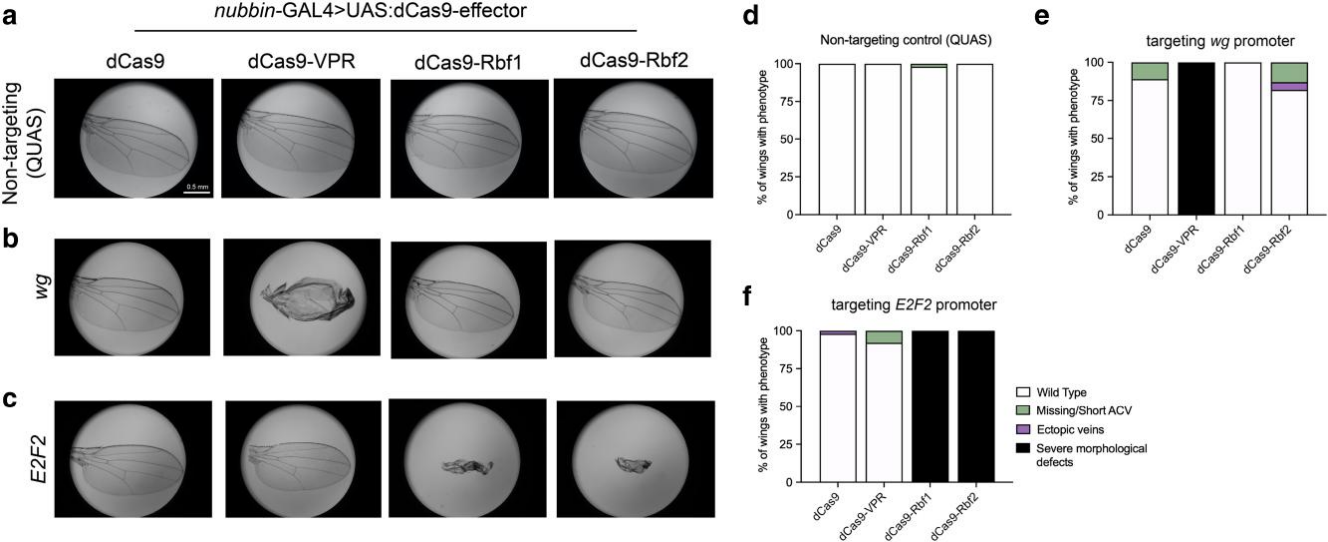
Targeting dCas9-Rb chimeras to endogenous promoters leads to gene-specific effects in the wing. a–c) Wings (*n* = 100) were dissected from adult flies for phenotype analysis after dCas9-effectors were targeted to endogenous gene promoters in L3 wing discs. Representative images from each cross are shown. a) A non-targeting gRNA control (QUAS) demonstrates that expression of effectors not targeted to any locus on the genome do not cause adult wing phenotypes. b) Targeting the *wg* promoter with VPR leads to severe developmental defects, but Rb effects on this promoter are mild to non-existent. c) Targeting the *E2F2* promoter with Rbf1 and Rbf2 leads to severe morphological defects that are Rb-specific, confirming the feasibility of the dCas9-Rb fusions in vivo in Drosophila. d–f) Quantification of the wings analyzed in a–c). Values are indicated as a proportion of the total number of wings. d) Quantification of images in a). Background effects are essentially non-existent when expressing the dCas9-effectors with a non-targeting gRNA. e) Quantification of images from crosses depicted in b). dCas9-VPR has a severe phenotypic effect on *wg*, due to *wg* overexpression, as previously reported (Ewen-Campen *et al*. 2017). Minor phenotypes are observed in ∼20% of wings with dCas9-Rbf2. f) Quantification of images from crosses depicted in c). Effects of targeting the *E2F2* promoter are severe with both Rb paralogs, and the phenotype is fully penetrant. Legend in f) is shown for all graphs.

Motivated by the successful application of CRISPRi in other model systems, we created novel dCas9 fusions to the Drosophila Rb paralogs, Rbf1 and Rbf2. To assess the activity of retinoblastoma paralogs on endogenous target genes in their native chromatin context in flies, we expressed FLAG-epitope-tagged dCas9-Rbf1, dCas9-Rbf2, or dCas9 alone in the third instar larval (L3) wing imaginal disc (Fig. 1a). The wing imaginal discs are sensitive to genetic changes during development; misregulation of genes that are vital for wing development often lead to morphological alterations that are readily observable in adult wings. Thus, we employed a screening strategy based on easily observable wing phenotypes to efficiently test this CRISPRi system in vivo. First, we created homozygous transgenic fly lines expressing *nubbin*-GAL4 and one of the UAS:dCas9-Rb effectors (Fig. 1b). The *nubbin*-GAL4 > UAS:dCas9-Rb flies were then crossed to gRNA fly lines, obtained from the Drosophila Research & Screening Center (DRSC). The gRNA flies are homozygous lines that express 2 tandem gRNAs targeting a single gene's promoter, within 400 bp of the gene's annotated TSS (Zirin *et al*. 2020). The progeny, expressing a single copy of each of the 3 transgenes (*nubbin*-GAL4, dCas9-effector, and gRNAs), were used in experiments described here (Fig. 1c, Supplementary Fig. 1; see Materials and methods). We also employed the dCas9-VPR chimera, which functions as a strong activator in Drosophila, and has been used in CRISPRa studies in vivo (Lin *et al*. 2015; Ewen-Campen *et al*. 2017; Ortega-Yáñez *et al*. 2022). Control flies expressing non-targeting gRNAs (QUAS) had phenotypically wild type (WT) adult wings, indicating that expression of a single copy of each of the dCas9 effectors in the wing does not impact normal development (Fig. 2a). In agreement with known Rbf1 and Rbf2 overexpression phenotypes in the fly wing, flies with 2 copies of the *nubbin*-GAL4 and UAS:dCas9-effectors (without any gRNAs) exhibited modest adult wing phenotypes such as notching on the wing margin, an initial indication that Rb function is not abrogated due to fusion to dCas9 (data not shown; Elenbaas *et al*. 2015).

### Gene-specific effects of Rbf1 and Rbf2 recruitment by dCas9

To gain insight into gene-specific transcriptional effects of Rb paralogs, we recruited dCas9-Rb effectors to the promoters of 2 genes, *wg* and *dpp*, which are key in regulating wing development. Furthermore, these genes are responsive to dCas9-VPR-mediated transcriptional activation using CRISPRa (Lin *et al*. 2015; Ewen-Campen *et al*. 2017). We reasoned that repression of these genes by dCas9-Rb targeting may produce a wing phenotype, as inhibition of either gene causes severe developmental defects in the wing (reviewed in Swarup and Verheyen 2012; Bosch *et al*. 2017). As previously reported, we found that the dCas9-VPR activator produces clear morphological defects when targeted to *wg* or *dpp–*an effect that was dependent on the activation domain, as dCas9 alone does not cause a similar phenotype (Fig. 2b and e, Supplementary Fig. 2a and c; Ewen-Campen *et al*. 2017). Targeting the *wg* promoter with the dCas9-Rb effectors did not produce a severe phenotype, although some mild effects are noted with dCas9-Rbf2 (Fig. 2b and e). In contrast, targeting both dCas9-Rbf1 and dCas9-Rbf2 to *dpp* leads to 20–30% of the wings with mild phenotypes, although this is less penetrant than effects of targeting dCas9 alone, which we discuss later (Supplementary Fig. 2a and c).

To determine whether we could identify promoters with Rb-specific effects, we targeted an additional 26 genes with diverse cellular functions (Table 1). The genes were selected based on established binding profiles of Rbf1 and Rbf2, and whether they are responsive to overexpression of the Rb paralogs in different tissue types (Wei *et al*. 2015; Mouawad *et al*. 2019; Mouawad *et al.* unpublished). Among the selected genes, recruiting dCas9-Rbf1 or dCas9-Rbf2 to the *E2F2* promoter led to severe morphological defects that surpassed the phenotypes from targeting any of the other genes (Fig. 2c and f). This phenotype was Rb-specific and not observed with dCas9 or dCas9-VPR recruitment, making it an ideal locus for further investigation into potential intrinsic differences between the 2 Rb paralogs.

In addition to *E2F2*, recruitment of Rb proteins to a dozen of the candidate promoters led to a visible adult wing phenotype (Supplementary Fig. 2, Table 1). On some promoters, recruitment of dCas9-tethered Rbf1 and Rbf2 generated similar wing phenotypes (*E2F2*, *Acf*), while on others, phenotypically distinct effects were observed (*InR*, *mcm6*, *Pex2*) (Fig. 2, Supplementary Fig. 2). For instance, on the *mcm6* promoter, dCas9-Rbf1 recruitment led to ∼70% of the wings with a phenotype that can be described as a combination of supernumerary bristles and short ACVs, while dCas9-Rbf2 recruitment led to a quarter of wings with ectopic veins and another quarter with short ACV, indicating possibly different transcriptional effects on this locus, with differing morphological consequences (Supplementary Fig. 2e). On the *InR* promoter, dCas9-Rbf2 effects were much greater than dCas9-Rbf1 effects, although they were indistinguishable from dCas9 alone. dCas9 effects were also observed on *mcm6*, *dpp* and *Acf*; we hypothesize that on these promoters, dCas9 recruitment is sufficient to block the RNA polymerase initiation or to occlude the binding of endogenous transcriptional activators or repressors, as has been suggested for a handful of other Drosophila genes (Ghosh *et al*. 2016; Huynh *et al*. 2018; Aguilar *et al*. 2023).

Over half of the targeted promoters lacked a visible phenotypic response to Rb recruitment. In some cases, transcriptional perturbation of specific genes may be irrelevant for wing pouch development, or the perturbation may have been too subtle. Alternatively, the positioning of the gRNAs may not have been ideal. Nonetheless, the dozen gene targets with visible phenotypes indicated that the CRISPRi method is useful in identifying genes with strong or weak effects (Table 1). To further investigate transcriptional impacts and potential differences in magnitude of effects mediated by the 2 Rb paralogs, we further explored the *E2F2* locus, which exhibited the most striking phenotype.

### Effect of CRISPRi on the *E2F2/Mpp6* bidirectional promoter in wings and in ovary

**Fig. 3.**
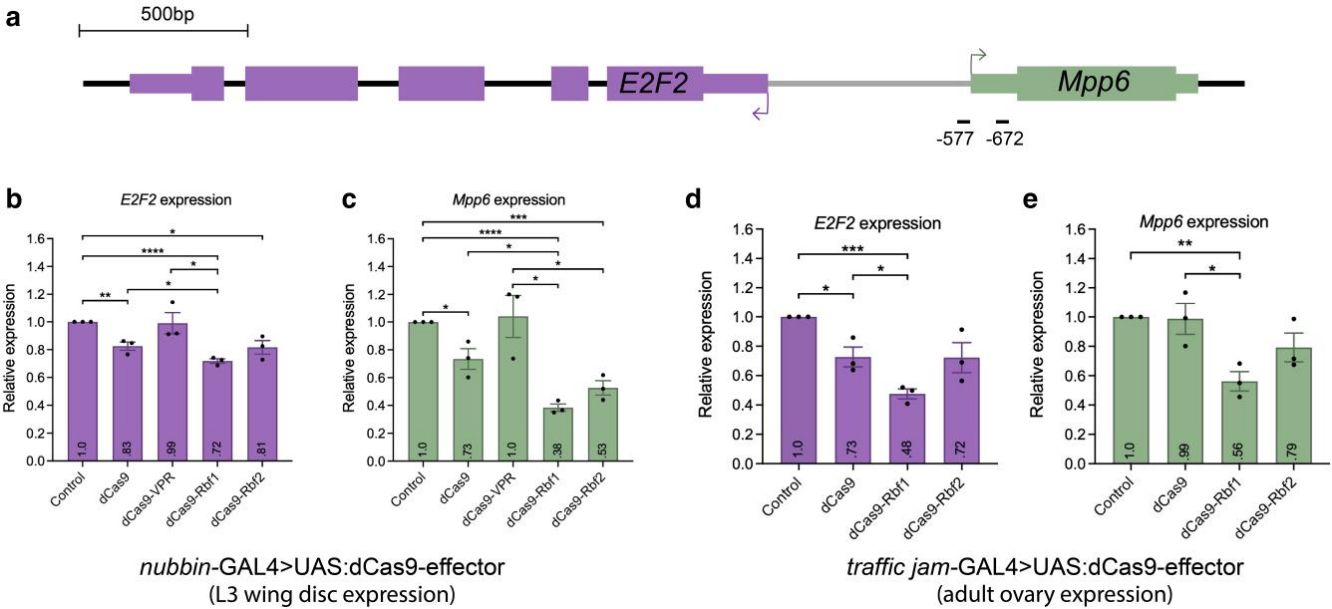
Rbf1 and Rbf2 recruitment to the *E2F2/Mpp6* promoter leads to Rb-specific repression. a) Diagram of the *E2F2* gene, which shares its promoter with *Mpp6* as a divergently paired gene. gRNA binding sites on the promoter of *E2F2*, which overlaps with the promoter of *Mpp6*. Horizontal lines below the genes are the locations of the *E2F2* gRNAs, with approximate distance from the indicated *E2F2* TSS. b, c) Expression level of *E2F2* and *Mpp6* measured in L3 wing discs after dCas9-effector recruitment, using RT-qPCR. Control samples are dCas9 flies crossed to a non-targeting gRNA fly line (QUAS). b) Rbf1 and Rbf2 significantly repress *E2F2* expression. dCas9 alone leads to some modest repression, while the VPR activator has no significant effect on expression of *E2F2* or *Mpp6*. c) Rbf1 and Rbf2 significantly repress *Mpp6* expression, to a greater degree than observed for *E2F2*. Effects are significantly greater than by dCas9 alone. d, e) Expression level of *E2F2* and *Mpp6* measured in ovaries after dCas9-effector recruitment, using RT-qPCR. d) *E2F2* is significantly repressed by dCas9-Rbf1, which is greater than dCas9-Rbf2 and dCas9 effects. e) *Mpp6* is significantly repressed by dCas9-Rbf1, and dCas9-Rbf1 appears to be a better repressor than Rbf2, although the difference is not statistically significant. For b–e), values in the bars indicate the average expression, error bars are SEM, and asterisks denote statistically significant differences: *P < 0.05, **P < 0.01, ***P < 0.001, ****P < 0.0001.

*E2F2* encodes a transcriptional repressor in Drosophila that regulates cell cycle progression (Cayirlioglu *et al*. 2001; Frolov *et al*. 2001). In the developing embryo, Rbf1 and Rbf2 bind near the TSS of *E2F2*, suggesting Rb proteins may directly regulate this gene (Table 1; Wei *et al*. 2015). To determine the magnitude of repression by each paralog, we measured the expression level of *E2F2* in the L3 wing discs after targeting dCas9-Rbf1 or dCas9-Rbf2 to its promoter. dCas9-Rbf1 targeting led to ∼30% repression of *E2F2*, while dCas9-Rbf2 targeting led to ∼20% repression, which was a similar level of repression as mediated by dCas9 alone (Fig. 3b). Thus, in this context, Rbf1 appears to be a modestly better repressor than Rbf2. The modest repression by dCas9, presumably via steric effects, is alleviated by VPR recruitment (Fig. 3b). Recruitment of dCas9-Rbf2 or dCas9 altered gene expression to a similar extent; yet, these 2 proteins had dramatically different effects on wing development (Fig. 2c). We explored whether the differences may reflect actions of the repressors on a neighboring gene.

Indeed, *E2F2* exists as a divergently-paired gene (DPG), sharing its promoter with the nearby *Mpp6* gene. DPGs, comprising ∼30% of Drosophila genes, are 2 genes that are transcribed from opposite strands and whose TSSs are found <1 kb apart (Yang and Yu, 2009). The *E2F2* tandem gRNA fly line used here expresses gRNAs for which the binding sites overlap with the predominant TSS of *Mpp6* (Supplementary Fig. 3). We measured the expression level of *Mpp6* in the L3 wing discs and observed >60% repression of *Mpp6* by Rbf1 and ∼50% by Rbf2 (Fig. 3c). dCas9 alone had a modest magnitude of effect on *Mpp6*, much milder than what was measured by dCas9-Rbf2 targeting. Thus, we hypothesize that the severe morphological defect caused by targeting this locus is due to the action of the dCas9-effectors on the *Mpp6* TSS. *Mpp6* encodes a protein that is involved in maturation of 5.8S rRNA; the repression of this gene may have been sufficient to impact cell growth, thereby leading to the severe wing phenotype (Schilders *et al*. 2005).

Rbf1 and Rbf2 expression patterns overlap in early embryogenesis and in the third instar imaginal discs; yet, in the adult, Rbf2 expression is restricted to ovaries, while Rbf1 is expressed in most tissue types (flybase.org; Stevaux 2002; Keller *et al*. 2005; Stevaux *et al*. 2005). To determine whether Rbf1 and Rbf2 transcriptional effects differ in the ovary, we expressed the dCas9-Rb effectors using the *traffic jam*-GAL4 driver, which is expressed predominantly in the follicle cells of the adult ovary (Weaver *et al*. 2020; Fig. 1d). Using the same *E2F2* gRNA fly line, we recruited the dCas9-effectors to the *E2F2*/*Mpp6* bidirectional promoter. We found that Rbf1 was a better repressor of both genes than Rbf2 (Fig. 3d and e). Additionally, the Rbf1 effects were more pronounced on *Mpp6* than on *E2F2*, relative to the magnitude of dCas9 effects. Thus, in the ovary, differences in magnitude of effects by each Rb paralog were similar to those observed in the wing. Taken together, we identified a locus on which dCas9-Rb chimeras significantly repress gene expression, leading to a severe developmental defect of the wing, and found a promoter that is more responsive to Rbf1 than to Rbf2 at the level of transcriptional repression, with possible preference for TSS-proximal sites.

### Position-sensitive Rb paralog repression on the *E2F2/Mpp6* bidirectional promoter

**Fig. 4.**
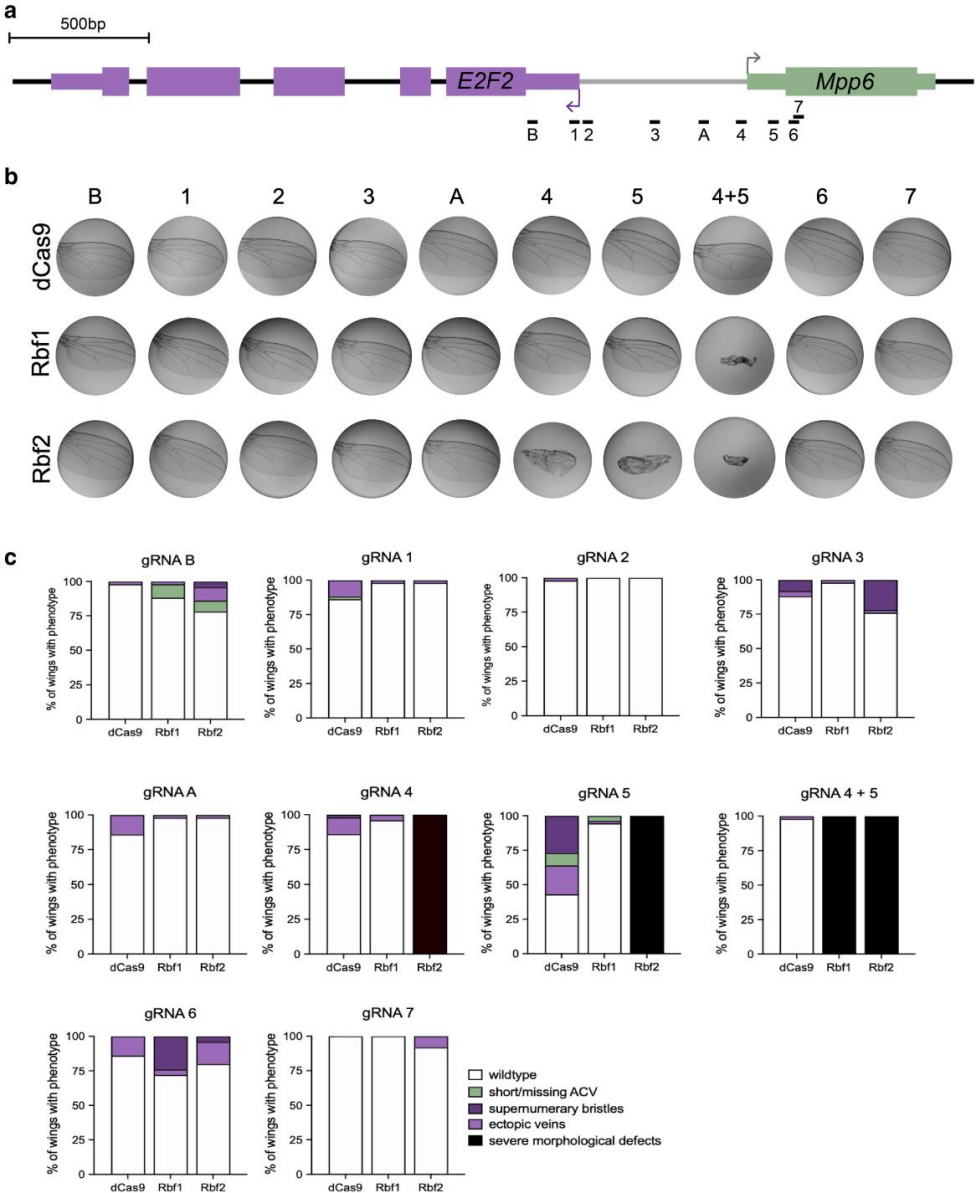
Position-sensitive Rb paralog effects on the *E2F2/Mpp6* bidirectional promoter. a) Schematic of single gRNAs targeting the 1 kb promoter between the *E2F2* and *Mpp6* transcribed regions. gRNA 4 is what is indicated as −577 in Fig. 3, and gRNA 5 is what is indicated as −672. 4 + 5 are the 2 tandem gRNAs used in Fig. 3. dCas9 effectors were recruited to these sites using one gRNA at a time. b) Adult wings (*n* = 50) were dissected after targeting by dCas9, dCas9-Rbf1, and dCas9-Rbf2. Images are representative wings. The severe morphological defect which is clear with 4 + 5 is only observed with gRNA 4 or 5 alone with Rbf2. Rbf1 targeting with the single gRNA 4 or gRNA 5 does not produce this severe effect. c) Quantification of the percentage of wings with particular phenotypes as indicated in the legend on the bottom right. Milder effects were observed from most sites, with supernumerary bristles or ectopic veins forming in a small proportion of wings.

We reasoned that the greater repression of *Mpp6* observed in the L3 wing discs could be due to the proximity of the gRNAs to this gene's TSS, and that moving the effectors away from *Mpp6* and toward *E2F2* would allow us to measure de-repression of *Mpp6*. To test this idea, we designed 9 gRNAs spanning the intergenic region and UTRs of *E2F2* and *Mpp6.* We generated new transgenic fly lines expressing each 1 of the 9 single gRNAs and crossed them to the *nubbin*-GAL4 > UAS:dCas9-Rb homozygous flies to generate triple transgenic flies (Fig. 4a).

Targeting the Rb effectors using single gRNAs on this locus led to a severe wing phenotype only with gRNA 4 and gRNA 5 (Fig. 4b and c), which are the 2 gRNAs that were used in tandem in the previous experiments (Figs. 2 and 3). Strikingly, from these 2 positions, only dCas9-Rbf2 recruitment causes a severe wing phenotype, while dCas9-Rbf1 does not, suggesting that recruitment of a single Rbf2 molecule to the *Mpp6* TSS is sufficient to generate this developmental defect. In contrast, Rbf1 may require cooperativity between 2 monomers to effect change. This is a clear instance of Rbf2 functioning as a more potent repressor than Rbf1. Moving the gRNAs away from the *Mpp6* TSS and towards the *E2F2* TSS results in loss of the severe wing phenotype, albeit with some minor phenotypes generated from targeting some of the other targeting sites on the promoter (Fig. 4c).

**Fig. 5.**
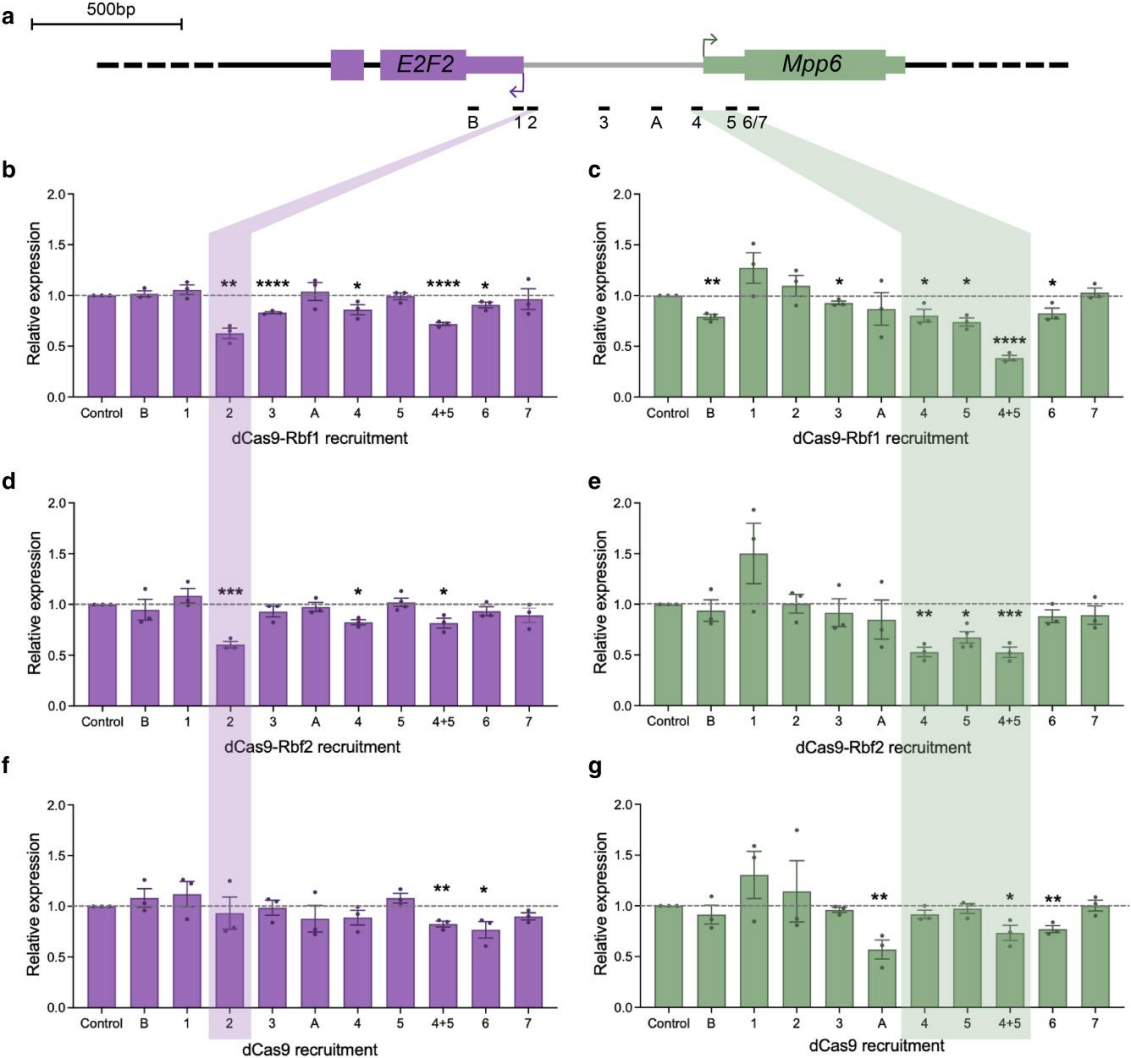
Repression of *E2F2* and *Mpp6* in a distance-dependent manner. a) Schematic of gRNAs designed here to target the *E2F2/Mpp6* promoter. dCas9 effectors were recruited to these sites using one gRNA at a time, and L3 wing discs were dissected for RT-qPCR analysis. b) Expression of *E2F2* after recruitment of dCas9-Rbf1 to each gRNA site. Position 2 is highlighted, where the greatest repression was measured (∼40%). c) Expression of *Mpp6* after recruitment of dCas9-Rbf1 to each gRNA site. Positions 4 and 5 are highlighted, where the greatest repression was measured. Rbf1 is a better repressor when 2 molecules are recruited to the *Mpp6* TSS. d) Expression of *E2F2* after recruitment of dCas9-Rbf2 to each gRNA site. Position 2 is highlighted, where the greatest repression was measured (∼40%), identical to what was measured by dCas9-Rbf1. e) Expression of *Mpp6* after recruitment of dCas9-Rbf2 to each gRNA site. Positions 4 and 5 are highlighted, where the greatest repression was measured. Whether a single molecule or 2 molecules of Rbf2 are recruited does not make a big difference in magnitude of repression (∼50%). f) Expression of *E2F2* after recruitment of dCas9 to each gRNA site. dCas9 is not able to repress this gene from most sites. g) Expression of *Mpp6* after recruitment of dCas9 to each gRNA site. dCas9 is not able to repress this gene from most sites, aside from a few. Error bars indicate SEM, and *P < 0.05, **P < 0.01, ***P < 0.001, ****P < 0.0001.

We next measured the expression level of both *E2F2* and *Mpp6* from each of these gRNA positions, to determine the ability of the Rb paralogs to repress gene expression from different distances relative to the 2 genes' start sites (Fig. 5). For *Mpp6*, positioning the chimeric proteins at position 4 or 5 had the greatest effect on transcription, with Rbf2 causing up to 50% reduction, while a modest ∼20–25% decrease in transcript levels were measured after Rbf1 targeting, with no phenotypic changes (Figs. 4, 5c and e). We posit that significant wing developmental defects emerge only after a certain threshold of *Mpp6* repression is achieved. Moving the Rb paralogs away from the *Mpp6* TSS and closer to *E2F2*, *Mpp6* is de-repressed, consistent with these Rb paralogs functioning in a distance-dependent manner, exhibiting optimal activity when present near the *Mpp6* TSS (Fig. 5c and e).

For *E2F2*, we measured the greatest reduction in transcript levels from position 2, directly 5′ of the major TSS (Fig. 5b and d). Here, recruitment of either Rbf1 or Rbf2 led to ∼40% repression, but is not associated with a severe wing phenotype. This result supports the hypothesis that the phenotype from position 4/5 is a consequence of *Mpp6* repression, rather than the milder *E2F2* repression measured from that site. More 3′ positions 1 and B were not ideal for repression of *E2F2*. Intriguingly, a single Rbf1 molecule was able to mediate significant repression of *E2F2* from position 2, but exhibited only very subtle effects on transcription of *Mpp6* from a similar TSS-proximal position. These data indicate that innate promoter structure and/or chromatin architecture may impact inherent repression potential, consistent with the different effects noted in our screen of 28 loci. Taken together, for both *E2F2* and *Mpp6*, optimal repression by Rb paralogs is seen at promoter-proximal sites, consistent with a short-range mechanism of repression.

### Position-sensitive Rb paralog repression in cell culture

**Fig. 6.**
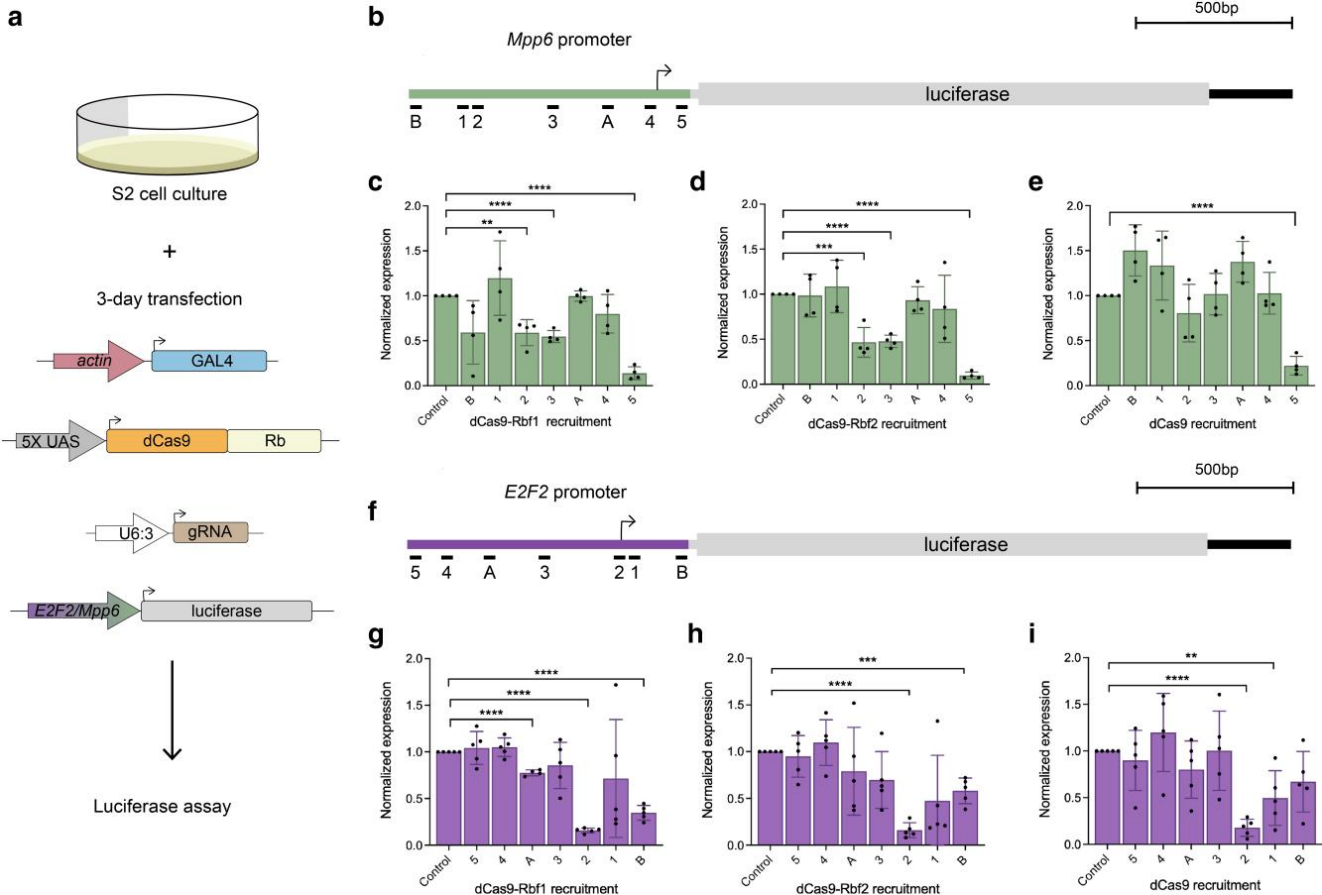
Rb paralogs have similar transcriptional effects on *E2F2* and *Mpp6* reporters in S2 cells. a) For all experiments described here, S2 cells were transfected with *actin*-GAL4, one of the dCas9 effectors, a single gRNA, and a luciferase reporter. b) Schematic of the *Mpp6*-luciferase reporter that was designed to be regulated by the *Mpp6* promoter. gRNAs are identical to those used in vivo, but lack gRNA 6 and 7, as they bind within the protein coding sequence not present in the reporter. c) dCas9-Rbf1 recruitment to the *Mpp6* reporter significantly represses luciferase expression from position 2 and 3 (50%). Position 5 repression appears to represent a nonspecific steric hindrance, as it is seen with dCas9 alone e). d) dCas9-Rbf2 has a similar pattern and degree of repression of this reporter as dCas9-Rbf1. e) dCas9 has little effect on this promoter aside from position 5, which suggests steric hindrance from downstream of the TSS. f) Schematic of the *E2F2*-luciferase reporter that was designed to be regulated by the *E2F2* promoter. Here, the gRNAs are in opposite orientation from what is shown in a), as this is the same genomic region, but on the opposite strand. g) dCas9-Rbf1 recruitment to the *E2F2* reporter significantly represses luciferase expression from position A (25%) and position B (60%). Recruitment to position 2 causes the same level of repression as dCas9 alone, suggesting steric hindrance from this site, as seen in i). h) dCas9-Rbf2 has a similar pattern and degree of repression of this reporter as dCas9-Rbf1. i) dCas9 has little effect on this promoter, aside from position 2 and 1, which suggests that the *E2F2* promoter is more sensitive than the *Mpp6* promoter to recruitment of dCas9. Error bars indicate SEM, and *P < 0.05, **P < 0.01, ***P < 0.001, ****P < 0.0001. Expression is normalized to the empty gRNA vector control.

Previous Drosophila Rb studies have relied on measuring either global changes in gene expression after Rb perturbations or changes in expression of reporter genes in cell culture (Dimova *et al*. 2003; Stevaux *et al*. 2005; Payankaulam *et al*. 2016; Mouawad *et al*. 2019, 2020; Zappia *et al*. 2019). Even the recent high-throughput analysis of Drosophila Rb paralog activity has relied on uncovering the differential impact on enhancers by using non-integrated reporters in S2 cells (Jacobs *et al*. 2023). To compare our in vivo results to transient transfection assays in cell culture, we designed reporter genes in Drosophila S2 cells. We generated 2 luciferase reporters using the *E2F2* and *Mpp6* promoters, and recruited dCas9-Rb effectors across the promoters using the gRNAs designed here (Fig. 6a, b, and f).

On the *Mpp6*-luciferase reporter, we measured the strongest repression (∼50%) from gRNA positions 2 and 3, which bind at around −600 and −400 bp from the *Mpp6* TSS, respectively (Fig. 6c and d). The activity noted for gRNA3 in this cell-based transient assay contrasts with the lack of action in vivo, indicating that the gRNA is intrinsically capable of targeting the promoter, but other properties of the endogenous locus, such as chromatin structure, dictate repression function. We also measured significant repression (>75%) from position 5, an effect that was observed with dCas9 alone, suggesting steric hindrance from dCas9 recruitment 3′ of the TSS (Fig. 6e). In contrast to what was observed in vivo, Rbf1 and Rbf2 had an almost indistinguishable effect and could repress from sites distal to the TSS. Interestingly, the effects observed from targeting the dCas9 effectors to the *Mpp6* reporter are distinct from those observed on the *E2F2* reporter, which is driven by the same *cis*-regulatory region, but in the opposite orientation (Fig. 6f–i). For the *E2F2*-luciferase reporter, the nonspecific CRISPRi effect at gRNA 5 is absent, while gRNA 2, which overlaps the *E2F2* TSS, shows non-specific interference by dCas9 (Fig. 6i). Non-specific repression is also observed from positions 1 and B, which are immediately 3′ of the *E2F2* TSS. A clear distance-dependent effect of dCas9-Rb effectors is seen on this reporter as well. Taken together, the range of action on the *Mpp6* and *E2F2* reporters suggests that Rb proteins can function from longer distances (∼600 bp) than was optimal for the native chromosomally integrated genes, and some of the distinctions between Rbf1 and Rbf2 effects in vivo are not observed with these reporter assays.

### Functional analysis of Rbf1 features necessary for repression

To better understand the features necessary for Rb repression activity, we generated dCas9 chimeras with 2 previously characterized Rbf1 mutants, Rbf1^ΔIE^, and Rbf1^Δpocket^ (Supplementary Fig. 4a). The Rbf1^ΔIE^ mutant removes the IE, a ∼60 residue domain in the C-terminus that regulates the stability of Rbf1 (Acharya *et al*. 2010; Sengupta *et al*. 2015). The Rbf1^Δpocket^ mutant removes the central pocket domain (∼350 residues), which is critical for Rb interactions with E2F proteins (Acharya *et al*. 2010).

One feature that has remained an enigma is the IE, which is conserved in the mammalian p130 and p107 proteins, but is non-existent in the fly Rbf2 and poorly conserved in mammalian Rb proteins; the presence or lack of the IE may distinguish the paralogs' repression abilities from one another (Acharya *et al*. 2010; Sengupta *et al*. 2015). Rbf1^ΔIE^ is still able to co-immunoprecipitate with Drosophila E2F proteins, suggesting that promoter recruitment is not impacted; yet, it is unable to repress many reporter genes (Acharya *et al*. 2010; Raj *et al*. 2012a, 2012b). Still, there are certain reporter genes on which this mutant maintains its ability to function as a repressor, putting into question the significance of the IE in regulation of gene expression, and whether this domain is required for promoter targeting or for intrinsic repression activity (Raj *et al*. 2012a). We created a dCas9-Rbf1^ΔIE^ chimera and recruited it to the *E2F2/Mpp6* promoter in wing discs using gRNAs 4 + 5 (Supplementary Figs. 1 and 4a). Recruitment to this locus led to the same phenotypic effect in the wing and same magnitude of repression as the WT Rbf1 effector (Supplementary Fig. 4b–e). These results are corroborated by our reporter assay in S2 cells, where dCas9-Rbf1^ΔIE^ can repress the *Mpp6*-luciferase reporter to the same extent as dCas9-Rbf1 (Supplementary Figs. 4f and 6c).

While the IE seems to be dispensable in certain contexts, the pocket domain is absolutely necessary for E2F binding, as has been demonstrated extensively in many model organisms (Dick 2007). The pocket domain is used to recruit Rb to the transactivation domain of E2F activators, and itself recruits a variety of chromatin modifying complexes to mediate gene repression (Zhang and Dean 2001). In the fly, an Rbf1^Δpocket^ mutant is completely unable to repress a canonical Rbf1 target, *PCNA* (Acharya *et al*. 2010; Raj *et al*. 2012b). Yet, mutations in the LxCxE binding motif found in the pocket, or in pocket domain residues required for interaction with E2F proteins, do not abolish mammalian Rb protein function in vivo, calling into question the requirement of the conserved pocket domain for the entirety of Rb activities, and focusing attention on possible actions of the N- and C-terminal domains (Sellers *et al*. 1998; Sun *et al*. 2006; Cecchini *et al*. 2014). To test the significance of the pocket domain for intrinsic repression activity in our system, we generated a dCas9-Rbf1^Δpocket^ chimera (Supplementary Figs. 1 and 4a). Strikingly, recruitment of Rbf1^Δpocket^ to the *E2F2/Mpp6* promoter using gRNAs 4 + 5 resulted in the same severe phenotype as dCas9-Rbf1. Additionally, this mutant was capable of repressing both *E2F2* and *Mpp6* to the same magnitude as the WT protein (Supplementary Fig. 4b–e). These results were also corroborated in cell culture, where the pocket mutant functioned similarly to WT Rbf1 on the *Mpp6*-luciferase reporter, suggesting that Rbf1^Δpocket^ retains functionality (Supplementary Fig. 4g). These data point to an intrinsic repression activity separate from the pocket domain of Rbf1.

## Discussion

### Differences in repression ability of Rb paralogs

Until now, there has been relatively little information on whether structural divergences between Rbf1 and Rbf2 impact inherent transcriptional activity. For instance, Rbf1 and Rbf2 have different abilities in repressing specific gene promoters, but this may be strictly a function of differences in promoters they bind to, rather than inherent differences in ability to repress (Wei *et al*. 2015; Mouawad *et al*. 2019). Some of the earliest molecular biology studies pointed to invariant mechanisms of repression by Rb paralogs. For instance, GAL4-Rb paralog chimeras act in a similar manner on reporter genes (Bremner *et al*. 1995; Ferreira *et al*. 1998; Meloni *et al*. 1999). Chimeras interchanging the mammalian Rb and p107 pocket domains retain function as GAL4 fusions, indicating overlapping gene-regulatory abilities (Chow *et al*. 1996). Thus, many functional studies have concentrated on the similarities between the proteins. However, structural innovations among the Rb paralogs may impact actual repression potential, once bound to a locus. The recent study by Jacobs *et al.* (2023) reported an extensive comparison of corepressors, including Rbf1 and Rbf2, in their activity to modulate Drosophila enhancers in a high-throughput assay. They found that Rbf1 and Rbf2 chimeras, while not producing identical patterns of activity, were much more similar to each other than to other corepressors, including CtBP, SIN3 and CoREST, providing some evidence for both functional conservation as well as divergence (Jacobs *et al*. 2023). Thus, the mechanistic significance of Rb paralogy and diversification in several metazoan lineages remains unclear.

In this study, we set out to compare the potential for gene repression by Rbf1 and Rbf2, bypassing their differential recruitment to gene promoters through tethering them to dCas9 and recruiting them to the same genomic locations in vivo. It is notable that our assays of these chimeric fusions uncovered similarities but also important functional distinctions, particularly when assayed on endogenous targets. From our survey of effects at the transcriptional level, we find that most frequently, Rbf1 is a more potent repressor, although Rbf2 is more effective in specific contexts. For instance, only Rbf2 was able to cause the severe wing phenotype when targeting *E2F2/Mpp6* using single gRNAs in positions 4 or 5, possibly because of a greater ability to work as a single nucleation site for interactions with chromatin modifying factors; Rbf1 may have weaker interactions that are stimulated by positioning multiple Rbf1 molecules in close proximity on the promoter, as was the case with the enhanced repression using the tandem gRNAs 4 + 5. Future studies aimed at uncovering different inherent repression mechanisms by the Rb paralogs would benefit from measuring the impact on local histone modifications after dCas9-Rb recruitment to a locus, and identifying Rb interaction partners at the recruitment site, which may reveal different mechanisms of repression at specific loci.

### Towards the development of CRISPRi in the fly

Here, we present one of the first reports of a dCas9-repressor fusion in Drosophila. Simultaneously with this work, we generated dCas9-CtBP chimeras and showed that the alternatively spliced CtBP isoforms in the fly have different repression abilities on the *E2F2/Mpp6* locus (Raicu *et al*. 2024). Yet, in Drosophila, CRISPRi development has lagged behind that of CRISPRa, with few reports of success (Ghosh *et al*. 2016; Zirin *et al*. 2022; Aguilar *et al*. 2023; Raicu *et al*. 2024). A successful chimeric fusion of dCas9 to a repressor domain has not yet been identified for genome-wide gene repression in the fly, but our success in impacting select loci can serve as a stepping stone to guide future development.

Our study proved that Rbf1 and Rbf2 activity can be effectively deployed as part of a dCas9 chimeric repressor, with substantial levels of repression and measurable developmental effects in some cases. However, the specific gene- and position-specific effects noted in most cases indicate that these corepressors (and perhaps most corepressors, considering the corepressor-enhancer specificity noted in Jacobs *et al*. 2023) are not universal tools that will reliably disrupt gene expression in a promiscuous fashion, as one might desire for genome wide screening. To a certain degree, this may reflect the robustness of the wing developmental program and requirement of the selected genes for wing development post third instar larval stage. Alternatively, the diversity of cis-regulatory elements and trans-acting factors on each of these targeted promoters may play a role in permissivity of Rb-mediated repression, or the gRNA efficiency may not have been optimal. These possibilities have also been suggested for Drosophila CRISPRa experiments in which the VPR effector has no effect with certain gRNAs targeted to select promoters (Xia *et al*. 2023). For such applications, perhaps the nonspecific promoter-blocking action of dCas9 as described here, or Cas9 alone with truncated gRNAs as described in Auradkar *et al*. (2023) will be more suitable, with the proviso that such deployment lacks the finesse of partial gene deactivation via specific enhancer targeting, as has been demonstrated in mammalian cells with dCas9-KRAB fusions (Alerasool *et al*. 2020; Auradkar *et al*. 2023).

This study identified a more important aspect of repression biology; namely, the context-specific effects that are evident when Rb corepressors are introduced into diverse promoters and positions. The logic of *cis* regulation in eukaryotes invariably involves a high degree of combinatorial interactions amongst multiple proteins of the core machinery, chromatin, and regulatory transcription factors. A single binding site for a factor rarely evokes a measurable biological response, which provides an important level of buffering for biological systems to ignore the many non-regulatory physical interactions occurring with the genome and associated regulatory factors. In addition to providing a threshold to avoid unwanted regulatory effects, context-specificity for Rb and other corepressors likely opens the door to “soft repression” i.e. partial downregulation of targets that correspond to homeostatic adjustments of widely-expressed genes (Mitra *et al*. 2021). Many of the targets of Rbf2, for instance, appear to be genes involved in cell growth and growth control, whose overall expression might be tightly tuned by feedback signals (Wei *et al*. 2015; Mouawad *et al*. 2019). Thus, the quest for the golden fleece of a universal repressor may have instead brought us deeper appreciation for how the cell deploys its diverse regulatory machinery in subtle and powerful manners.

### Comparing effects on endogenous promoters vs on reporter genes

The CRISPRi approach also proved to be highly informative about the utility of transient reporter assays used in many functional studies of Rb biology and paralogy. By deploying identical gRNAs and CRISPRi effectors on the *E2F2* and *Mpp6* regulatory region in the developing wing and on reporter genes in cell culture, we found that the transiently transfected reporters faithfully reflected only certain properties of the regulation observed in vivo. Specific distance-dependent effects were not reproduced on the reporters, and a non-specific promoter-blocking action of the CRISPRi molecules was only found on the reporters, likely because of the less-defined chromatin state of these plasmid-borne genes. In addition, the stark difference between Rbf1 and Rbf2 in vivo was not recapitulated in cell culture, where Rbf1 and Rfb2 effects were almost identical. While transient reporters may not provide a complete picture of repression potential, they do remain a useful tool—these experiments provided a suitable test for the gRNAs that were inactive in the wing disc, such as gRNA 1, showing that the gRNA was at least capable of mediating steric hindrance in S2 cells. We argue that in the future, studies using CRISPRi in Drosophila will benefit from targeting endogenous loci, as was done here, to capture physiologically relevant effects of co-repressors such as Rb.

### Features necessary for repression

The dCas9 tethering approach also facilitated the use of mutants that could be directly compared with the wild-type Rbf1 protein in the same context. Rbf1 mutants have been extensively studied in Drosophila, with most experiments relying on global overexpression, with accompanying complexities due to pleiotropy (Acharya *et al*. 2010; Raj *et al*. 2012a; Zhang *et al.* 2014; Elenbaas *et al*. 2015). Overexpression of the Rbf1^ΔIE^ mutant in the wing disc is ineffective at global gene repression, compared with WT Rbf1 (Mouawad *et al.* unpublished). Yet, on a few reporter genes, it can still function to repress gene expression (Raj *et al*. 2012a). Thus, conflicting data has made it challenging to separate the function of the IE in promoter recruitment vs repression activity. Our results here, which show identical magnitude of repression by Rbf1^ΔIE^ and WT Rbf1 suggest that the IE is not required for transcriptional repression on the targeted promoter. We conclude that the inability of Rbf1^ΔIE^ to repress genes in previous Rbf1 overexpression assays is possibly due to a defect in its recruitment to DNA, rather than loss of inherent repression activity mediated by the IE. A key takeaway from the Rbf1^Δpocket^ mutant is that the N- and C-terminal domains of Rbf1 appear to be intact and functional in this context, perhaps allowing for interactions with cofactors required for repression. Indeed, the NTD resembles the cyclin folds of the pocket, and the CTD plays a role in interactions with E2F proteins; thus, in combination, the Rbf1 NTD and CTD may retain the ability to create a repression complex (Hassler *et al*. 2007). Interactions with cofactors have been reported outside of the pocket domain, making this a real possibility (Durfee *et al*. 1994; Hassler *et al*. 2007; Dick and Rubin 2013). Our study is the first to report on an Rb pocket deletion mutant with such potent repression ability. Future studies warrant studying the mutants tested here on additional endogenous loci to determine generalizability of the findings.

In conclusion, here we developed an in vivo CRISPRi system for targeting Drosophila Rb paralogs to gene promoters to directly query the effect of Rbf1 and Rbf2 on dozens of genes in the developing fly. We identified context-specific roles of Rb paralogs, as well as a short-range mechanism of gene regulation. These data hold promise for future development of CRISPRi in Drosophila, with potential widespread applications, as well as for understanding the significance of paralogy and the evolution of the conserved Retinoblastoma transcriptional corepressor proteins.

## Supplementary Material

jkae238_Supplementary_Data

## Data Availability

Plasmids generated in this study are available upon request. Fly lines generated in this study are available at Bloomington Drosophila Stock Center as indicated in the Supplementary Materials. The authors affirm that all data necessary for confirming the conclusions of the article are present within the article, figures, and tables. Supplemental material available at G3 online.
