# Supplementary material for: Retinoblastoma protein activity revealed by CRISPRi study of divergent Rbf1 and Rbf2 paralogs
Source: G3 (Bethesda). 2024 Oct 4;14(12):jkae238. doi: 10.1093/g3journal/jkae238 (PMC11631494; doi:10.1093/g3journal/jkae238)
Supplement: jkae238_Supplementary_Data [file jkae238_supplementary_data.docx]

**SUPPLEMENTARY MATERIAL FOR:**

# **Retinoblastoma protein activity revealed by CRISPRi study of divergent Rbf1 and Rbf2 paralogs**

Ana-Maria Raicu^1,2^, Patricia Castanheira^3^, David N. Arnosti^3*^

^1^ Cell and Molecular Biology Program, Michigan State University, East Lansing, MI, 48824, USA

^2^ Present address: Blavatnik Institute, Genetics Department, Harvard Medical School, Boston, MA, 02115, USA

^3^ Department of Biochemistry and Molecular Biology, Michigan State University, East Lansing, MI, 48824, USA

*corresponding author; arnosti@msu.edu

**Running head:** CRISPRi study of Drosophila Rb paralogs

**Keywords:** Retinoblastoma, CRISPRi, transcription, repression, Drosophila

**SUPPLEMENTARY FIGURES**

**
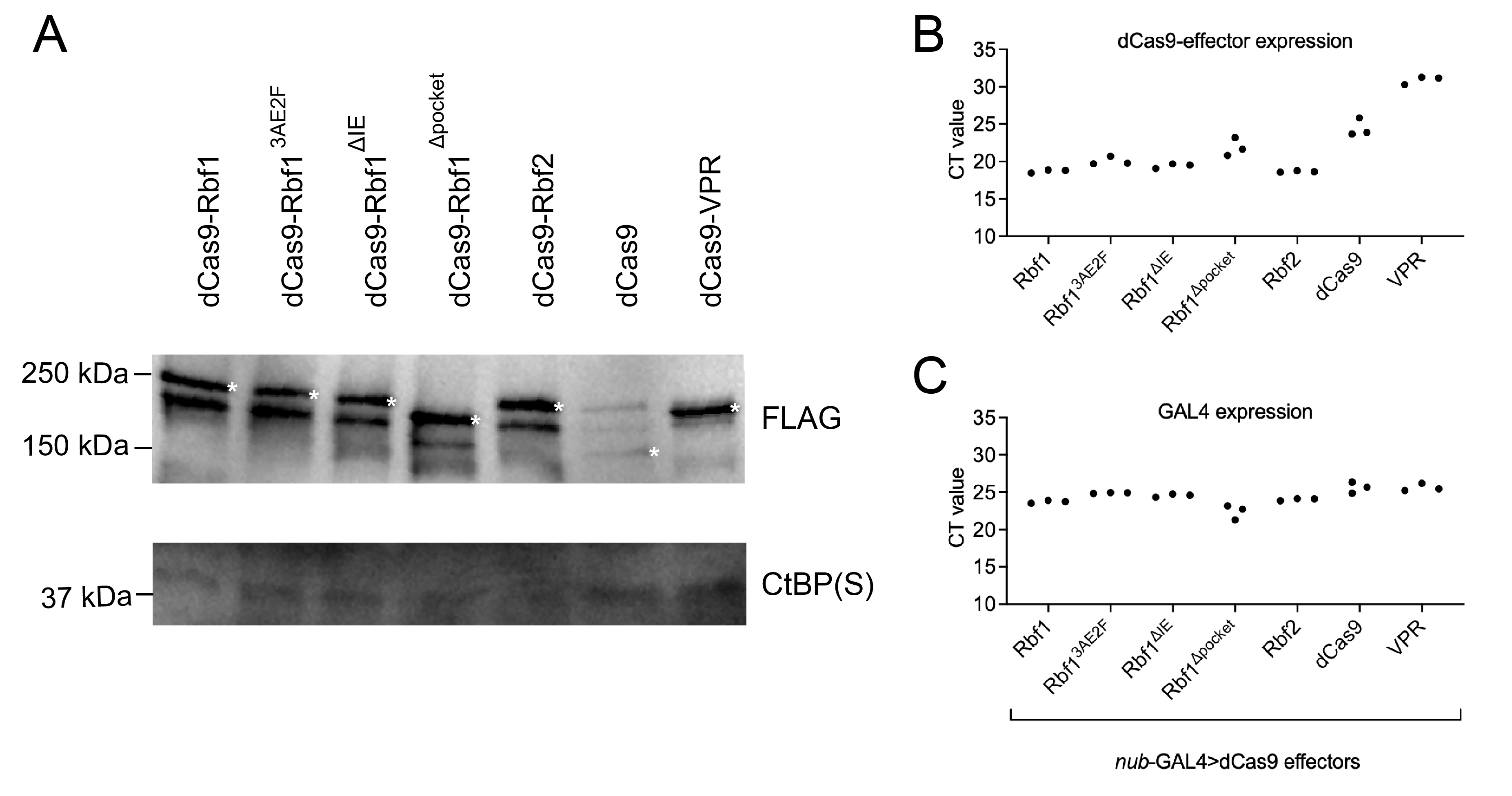
**

**Figure S1. Protein and mRNA expression of dCas9 effectors *in vivo*. A)** Western blot of dCas9 effectors expressed in L3 wing discs (in the triple transgene expressing flies, as shown in **Figure 1C**) shows similar levels of expression of effectors, aside from dCas9 alone, which is expressed at lower levels. Levels of endogenous CtBP(S) measured as loading controls. dCas9 effectors are detected with anti-FLAG antibody, and CtBP with anti-CtBP serum. White asterisks indicate the band of interest, with possible breakdown products below. **B)** mRNA levels of dCas9 effectors expressed in L3 wing discs were measured using RT-qPCR. Expression level (CT value) indicates that the effectors are expressed at relatively similar levels, aside from dCas9 and dCas9-VPR which are expressed at lower levels. **C)** mRNA levels of the *nubbin*-GAL4 transgene from L3 wing discs indicates that the GAL4 driver levels are similar from one genotype to the next, as expected.

**
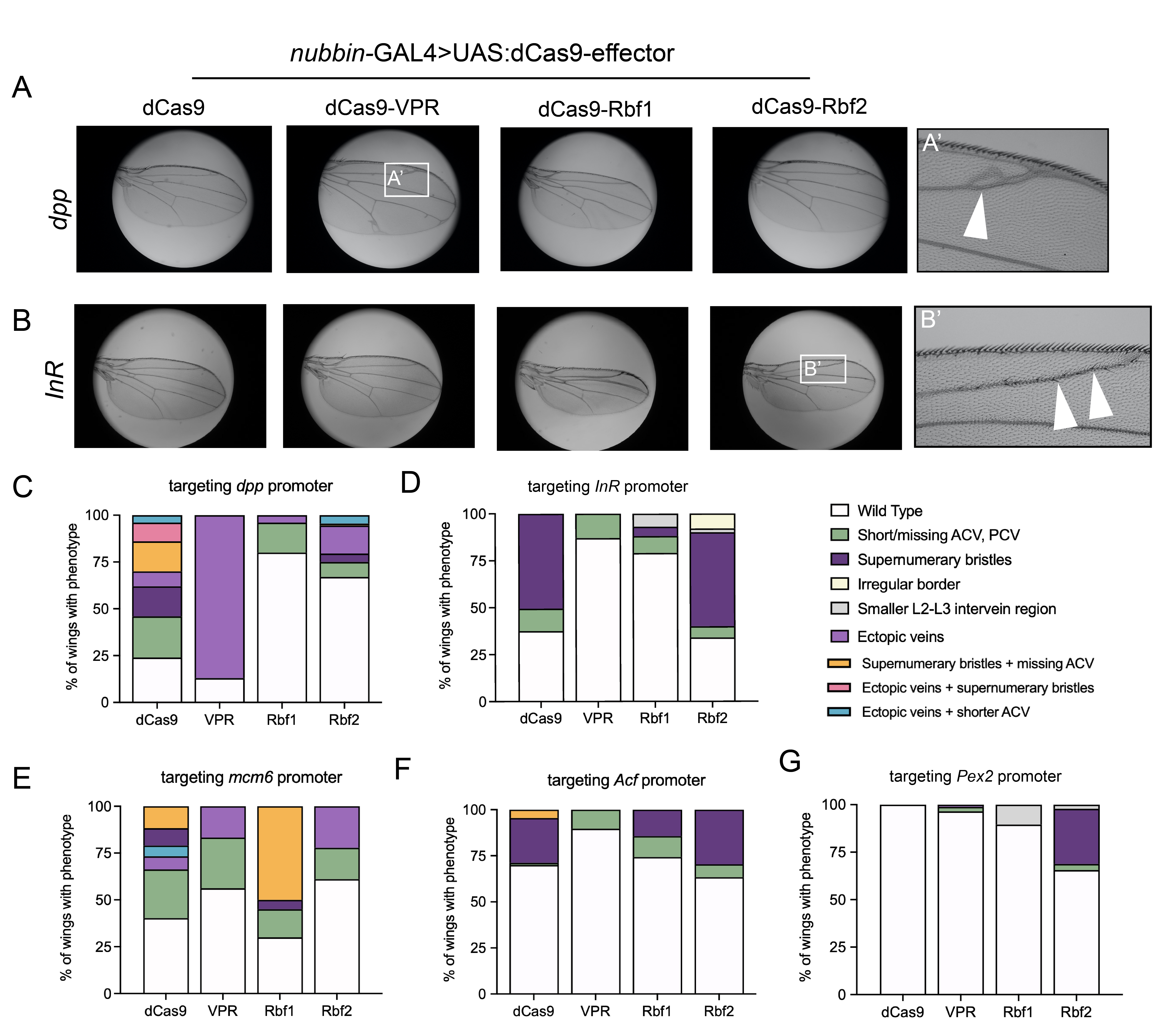
**

**Figure S2.** **Targeting Rbf1 and Rbf2 to endogenous gene promoters produces gene-specific effects.** Adult fly wings (n=100) were dissected after targeting dCas9-effectors as indicated in Figure 1. **(A)** Representative images from targeting the four effectors to the *dpp* gene promoter. A’ inset shows the ectopic vein phenotype with the white arrowhead. (**B)** Representative images from targeting the effectors to the *InR* gene promoter. B’ inset indicates the supernumerary bristle phenotype with the white arrowheads. **(C)** Quantification of images in (A). dCas9-VPR recruitment leads to ectopic vein formation in most wings. dCas9 alone leads to a phenotype that is more penetrant than dCas9-Rb effects. **(D)** Quantification of images in (B). Targeting *InR* leads to mild phenotypes with each effector, and clear dCas9 effects are also observed. Legend to the right is for all graphs. **(E)** Targeting *mcm6* caused a wide variety of phenotypes by all effectors, suggesting dCas9-induced changes, regardless of effector recruitment. **(F)** Targeting *Acf* causes mild phenotypes such as supernumerary bristle formation by dCas9, Rbf1, and Rbf2. **(G)** Targeting *Pex2* causes mild phenotypes that are Rb specific, and different between Rbf1 and Rbf2.

**
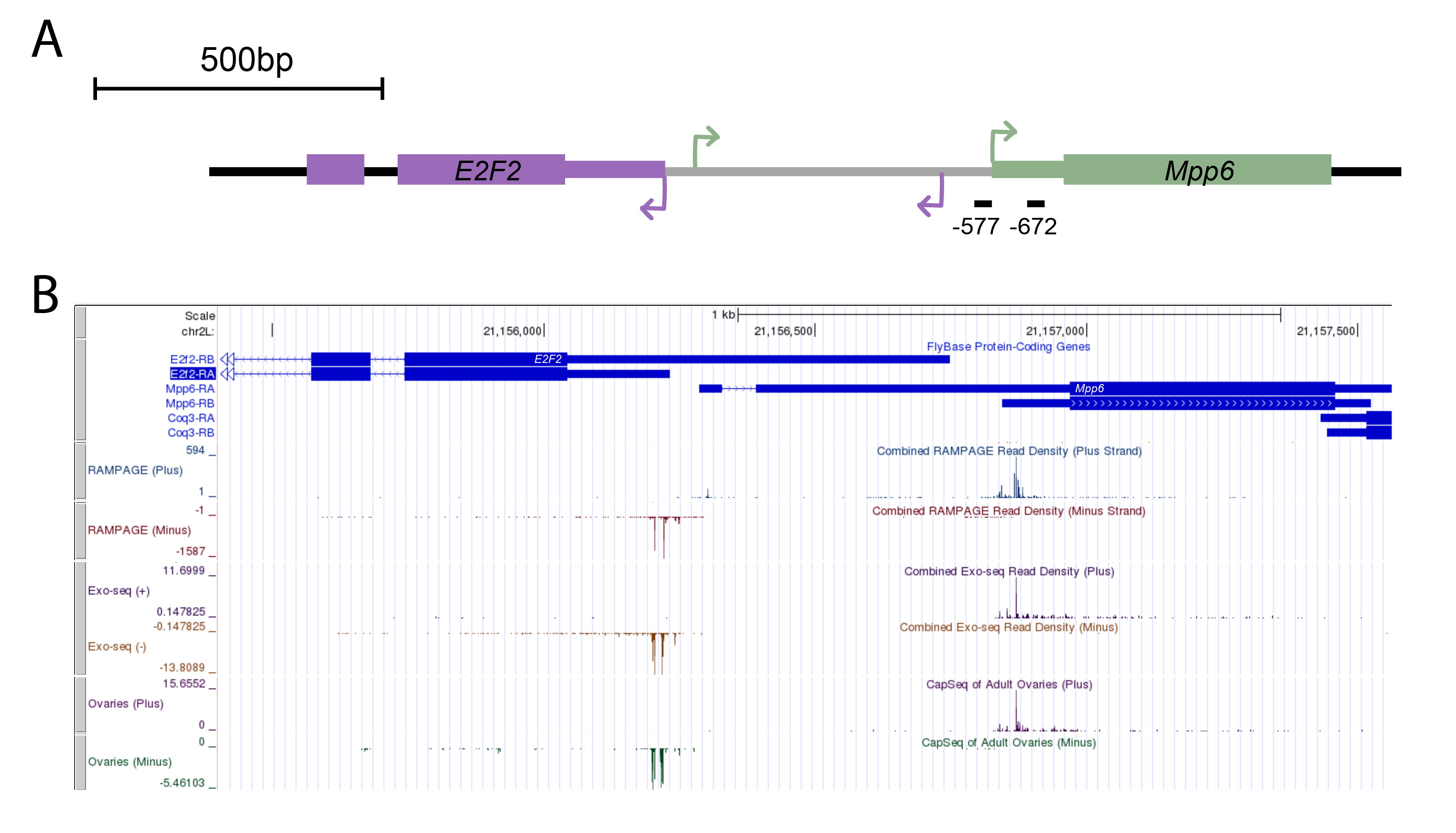
**

**Figure S3. *E2F2* and *Mpp6* exist as divergently-paired genes, and each predominantly uses a single, more proximal, TSS. A)** Schematic of the *E2F2/Mpp6* locus, with the two mapped TSSs (arrows) indicated for both genes. The tandem *E2F2* gRNA fly line expresses two gRNAs that bind close to the downstream TSS of *Mpp6* and the upstream TSS of *E2F2*. Here we indicate the gRNA binding sites (-577 and -672) relative to the downstream TSS of *E2F2*. **B)** ModENCODE snapshot of the *E2F2/Mpp6* locus, with RAMPAGE and Exo-seq tracks indicating the predominantly used TSS of both genes, including in ovaries. The downstream TSS is used for both genes.

**
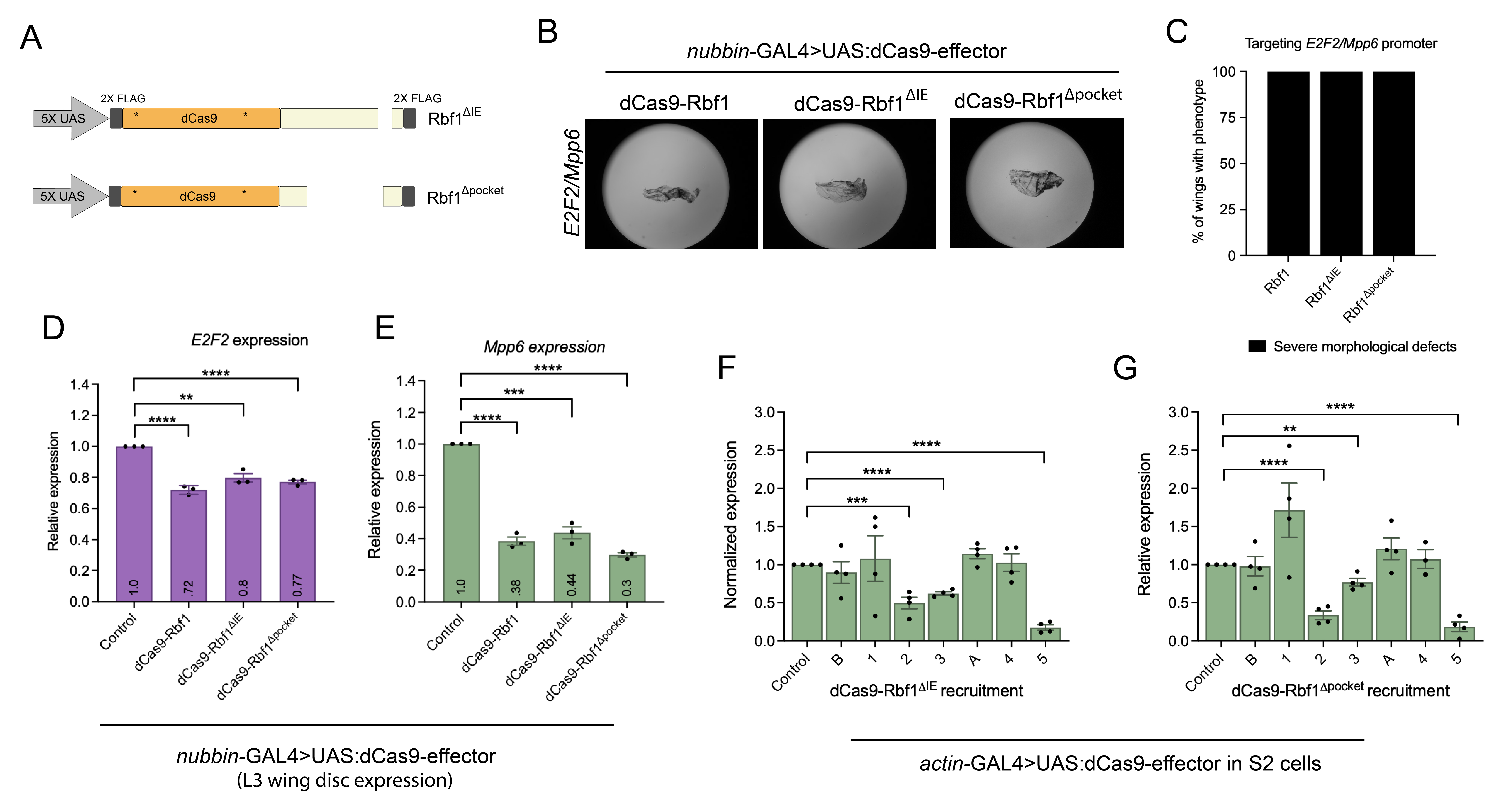
**

**Figure S4. Testing the domains required for Rbf1-mediated repression using dCas9. (A)** Schematic of two Rbf1 mutants used for dCas9 promoter targeting. ΔIE refers to removal of residues 728 to 786 from the C-terminus. Δpocket removes residues 376 to 728. **(B)** Recruitment to the *E2F2/Mpp6* locus using the tandem gRNAs 4 + 5 led to severe morphological defects with both mutants. **(C)** Proportion of wings with phenotypes after targeting Rbf1 mutants to position 4 + 5. **(D, E)** RT-qPCR data from targeting Rbf1 mutants to gRNA positions 4 + 5. *Mpp6* expression is repressed equally well by Rbf1^ΔIE^ and Rbf1^Δpocket^, as compared to WT Rbf1 (same data as in **Figure 3**), suggesting that the Instability Element and the pocket domain are not required for repression. **(F-G)** Effects of Rbf1^ΔIE^ and Rbf1^Δpocket^  mutants on the *Mpp6*-luciferase reporter. These mutants repress the reporter best from positions 2 and 3, which is identical to the WT Rbf1 effect (indicated in **Figure 6**). Repression at position 5 is due to steric hindrance, as dCas9 alone is able to mediate the same level of repression. Error bars indicate SEM, and * indicates p<0.05, ** is p<.01, *** p<.001, **** p<.0001.

**SUPPLEMENTARY TABLES**

**Table S1.** **Sequence of *E2F2* gRNAs designed in this study.**

| **gRNA** | **gRNA sequence(s)** |
| --- | --- |
| *E2F2* “B” | TATAGTCATCGAGTCGATTG |
| *E2F2* “1” | GAAAAAAATGACATAAATGG |
| *E2F2* “2” | GCAGTGCGCACGAAGAATAG |
| *E2F2* “3” | GGACAATAAACCTTAACGAA |
| *E2F2* “A” | AGTTGAGCTTGTTTGTCAGT |
| *E2F2* “4” | TTCAGCCTAGCTAGAAAACG |
| *E2F2* “5” | AAAACTAGGGCGAAACCATC |
| *E2F2* “6” | AGTCTCGGCTTTGATTTGGA |
| *E2F2* “7” | TCAAAGCCGAGACTTTCGCG |

**Table S2.** **Sequence of primers used for RT-qPCR analysis for each target gene.** F indicates Forward primer and R indicates Reverse primer. *CG8636*, *Rp49*, and *Rps13* were used as control genes.

| **Gene** | **Primers (Forward and Reverse)** |
| --- | --- |
| *CG8636* | F: GATCCGCTGCTAGATCCCAC  R: CCCTTGTACGGGCAGTTGA |
| *Rp49* | F: ATCGGTTACGGATCGAACAAGC  R: GTAAACGCGGTTCTGCATGAGC |
| *Rps13* | F: GGTCGTATGCACGCTCCT  R: CATCTGCGTTCAGTTTCAGC |
| *E2F2* | F: GACGAGGAAGTAGATATCAAGCG  R: TCAAAGAACCCATCCACATCG |
| *Mpp6* | F: GCTCGGTCATTCTGCTTTTG  R: CTCGGCTTTGATTTGGATGG |

**Table S3. Fly lines generated in this study.** The first six fly lines in the table express two copies of the dCas9-effector but with no GAL4 driver. The last three fly lines express two copies of the dCas9-effector and two copies of the *nubbin*-GAL4 transgene for L3 wing pouch specific expression.

| **BDSC Fly line #** | **Genotype** | **As referenced in this study** |
| --- | --- | --- |
| 99958 | *w[1118]; M{RFP[3xP3.PB] w[+mC]=UAS-dCas9-Rbf}ZH-86Fb* | UAS:dCas9-Rbf1 |
| 99959 | *w[1118]; M{RFP[3xP3.PB] w[+mC]=UAS-dCas9-Rbf2}ZH-86Fb* | UAS:dCas9-Rbf2 |
| 99960 | *w[1118]; M{RFP[3xP3.PB] w[+mC]=UAS-dCas9-Rbf.Deltapocket}ZH-86Fb* | UAS:dCas9-Rbf1^Δpocket^ |
| 99961 | *w[1118]; M{RFP[3xP3.PB] w[+mC]=UAS-dCas9-Rbf.DeltaIE}ZH-86Fb* | UAS:dCas9-Rbf1^ΔIE^ |
| 99963 | *w[1118]; M{RFP[3xP3.PB] w[+mC]=UAS-dCas9.FLAG}ZH-86Fb* | UAS:dCas9 |
| 602359 | *w[1118]; P{w[+mW.hs]=GawB}nubbin-AC-62; M{RFP[3xP3.PB] w[+mC]=UAS-dCas9-Rbf}ZH-86Fb* | *nubbin*-GAL4>UAS:dCas9-Rbf1 |
| 602360 | *w[1118]; P{w[+mW.hs]=GawB}nubbin-AC-62; M{RFP[3xP3.PB] w[+mC]=UAS-dCas9-Rbf2}ZH-86Fb* | *nubbin*-GAL4>UAS:dCas9-Rbf2 |
| 602361 | *w[1118]; P{w[+mW.hs]=GawB}nubbin-AC-62; M{RFP[3xP3.PB] w[+mC]=UAS-dCas9.FLAG}ZH-86Fb* | *nubbin*-GAL4>UAS:dCas9 |

**Table S4. Additional fly lines used in this study.**

| **BDSC Fly line #** | **Genotype** | **As referenced in this study** |
| --- | --- | --- |
| 25754 | *P{w[+mC]=UAS-Dcr-2.D}1, w[1118]; P{w[+mW.hs]=GawB}nubbin-AC-62* | *nubbin*-GAL4 |
| 3704 | *w[1118]/Dp(1; Y)y[+]; CyO/Bl[1]; TM2, e/TM6B, e, Tb[1]* | 3704 |
| 104055 (from DGRC) | [*y[*]*](http://flybase.org/search/symbol/FBgn/y) [*w[*];*](http://flybase.org/search/symbol/FBgn/w) [*P{w[+mW.hs]=GawB}*](http://flybase.org/search/symbol/FBtp/P%7BGawB%7D)[*NP1624*](http://flybase.org/search/symbol/FBti/P%7BGawB%7DNP1624) */* [*CyO,*](http://flybase.org/search/symbol/FBba/CyO) [*P{w[-]=UAS-lacZ.UW14}*](http://flybase.org/search/symbol/FBtp/P%7BUAS-lacZ.UW14%7D)[*UW14*](http://flybase.org/search/symbol/FBti/P%7BUAS-lacZ.UW14%7DUW14) | *Traffic jam*-GAL4 |
| 67055 | *w[*]; P{w[+mW.hs]=GawB}nubbin-AC-62; P{y[+t7.7] w[+mC]=UAS-3xFLAG.dCas9.VPR}attP2* | dCas9-VPR |
